# An economic evaluation of two PCR-based respiratory panel assays for patients admitted to hospital with community-acquired pneumonia (CAP) in the UK, France and Spain

**DOI:** 10.1186/s12890-023-02516-2

**Published:** 2023-06-21

**Authors:** Lisa Miners, Susie Huntington, Nathaniel Lee, Katy M. E. Turner, Elisabeth Adams

**Affiliations:** 1 Aquarius Population Health, Unit 29 Tileyard Studios, London, N7 9AH UK; 2 Hospital for Tropical Diseases, University College London Hospital NHS Foundation Trust, London, UK

**Keywords:** Community-acquired pneumonia, Diagnostics, Microbiology, COVID-19, Influenza, Legionella, Antimicrobial stewardship

## Abstract

**Background:**

On admission to hospital, patients with community-acquired pneumonia (CAP), undergo extensive diagnostic testing. Two high-throughput laboratory-based PCR panels which return a result in 5.5 hours (h) have been developed to test for pathogens commonly associated with upper (Respiratory 1 Panel) and lower (Respiratory 3 Panel) respiratory tract infections (GeneFirst, Oxford). These could replace multiple diagnostic tests currently used.

**Methods:**

An online survey, completed by senior clinicians in the UK, France and Spain, was used to collect data on the diagnostic testing of immunocompetent and immunocompromised adults admitted to hospital with CAP, including the cost of diagnostics. Data were used to inform a cost-comparison model. For each country, the average cost of diagnostic testing per patient was calculated separately for immunocompetent and immunocompromised patients.

The model compared three testing strategies with standard of care (SoC). In the Panel 1 strategy, the Respiratory 1 Panel was used for patients that would otherwise have tests which could be replaced by Respiratory 1 Panel, equivalent strategies for Respiratory 3 Panel and for both panels combined were assessed.

**Results:**

In total, 48 surveys were completed (UK = 17; France = 15; Spain = 16). Compared with SoC, the Panel 1 + 3 strategy was most favourable, resulting in cost savings for immunocompetent and immunocompromised patients respectively, of €22.09 (£18.50) and €26.12 (£21.88) in the UK, €99.60 and €108.77 in France and €27.07 and €51.87 in Spain.

**Conclusion:**

In all three countries, the use of these respiratory panels could reduce the average cost of diagnostics used for patients admitted to hospital with CAP.

**Supplementary Information:**

The online version contains supplementary material available at 10.1186/s12890-023-02516-2.

## Introduction

Community-acquired pneumonia (CAP) is a lung infection with an annual incidence rate in Europe of 1.6 to 10.6 per 1,000 adults [1, 2] and mortality rates between 5 and 14% [3]. Inpatient care for CAP admissions accounts for €5.7 billion annually across Europe [4]. CAP can be the result of a variety of bacterial and viral pathogens, with *Streptococcus pneumoniae* and respiratory viruses the most frequently isolated pathogens in CAP patients in Europe [4]. European guidelines [3, 5–9] recommend that, before microbiological investigations are requested, suspected CAP patients undergo initial clinical assessments including a chest X-ray, pulse oximetry, respiratory rate, and blood investigations. Patients are typically managed according to their symptom presentation. UK guidelines recommend that patients are treated with antibiotics within 4 h of presentation to hospital [5].

Although the UK Standard for Microbiology Investigations (SMI) recommends which diagnostic tests should be considered for CAP patients [10], diagnostic investigations are also guided by the patient’s immune status, their symptoms and the severity of their symptoms (as measured by the CURB-65 criteria used in many countries [11]) as well as the results of preliminary clinical investigations. Microbiological diagnostic tests typically include culture (of blood, sputum, or bronchoalveolar lavage (BAL) sample), urinary antigen tests and polymerase chain reaction (PCR) tests. For some pathogens, different hospitals or regions use different types of diagnostic tests according to access, preferences, capacity, cost and reimbursement. Microbiological investigations can be costly and may have lengthy turnaround times. In many cases, no causative pathogen is identified and 30–40% of CAP patients are discharged from hospital without a definitive microbiological diagnosis [2, 12].

Two PCR-based high-throughput respiratory panel assays with a 5.5-h turnaround have been developed for pathogens associated with upper (referred to as Respiratory 1 Panel, product code RESP003, GeneFirst, Oxford) and lower (Respiratory 3 Panel, product code RESP005, GeneFirst, Oxford) respiratory tract infections (Table 1). These can be used separately or together. The panels replace multiple diagnostic tests currently used and test for pathogens which might otherwise not be investigated or that would only be assessed following preliminary diagnostic testing. Hospital trusts, commissioners and laboratory managers will wish to consider the cost implications of implementing new respiratory panels into clinical practice. There is potential to reduce the overall laboratory costs and improve laboratory workflow by replacing several tests currently used with the respiratory panels. In addition, there may be clinical benefits to using the respiratory panels by increasing the number of cases where a microbiological diagnosis can be made and reducing the time to the most appropriate use of antibiotics.

**Table 1:** Pathogens detected by the high-throughput respiratory PCR panels

Target pathogens	Micro-organism type
Respiratory 1 Panel (upper respiratory pathogens)	
Influenza B	Virus
Influenza A	Virus
*Mycoplasma pneumoniae*	Bacteria
SARS-CoV-2 (two targets)^a^	Virus
Coronavirus group (229E, NKU1, NL63, OC43)^a^	Virus
Bocavirus	Virus
Rhinovirus	Virus
Metapneumovirus	Virus
HPIV group (HPIV 1, 2, 3 and 4)^a^	Virus
Adenovirus	Virus
Respiratory Syncytial Virus	Virus
Respiratory 3 Panel (lower respiratory pathogens)	
*Staphylococcus aureus*	Bacteria
*Bordetella pertussis*	Bacteria
*Moraxella catarrhalis*	Bacteria
*Legionella pneumophila*	Bacteria
*Coxiella burnetii*	Bacteria
*Pneumocystis jirovecii*	Fungi
*Streptococcus pneumoniae*	Bacteria
*Haemophilus influenzae*	Bacteria
*Mycoplasma pneumoniae*	Bacteria

*Abbreviations*: *HPIV* Human parainfluenza viruses, *PCR* Polymerase chain reaction, *SARS-CoV-2* Severe acute respiratory syndrome coronavirus 2

^a^The panel can detect the different types but the result does not specify which type is detected

The aim of this work was to calculate the current and expected costs per patient if the respiratory panels were to be implemented within the testing pathways for patients admitted to hospital with CAP in the UK, France and Spain. This will inform decision-makers who are evaluating the cost implications of adopting these tests in hospital laboratories.

## Methods

This section begins by describing the respiratory panels and listing which pathogens they detect (Table 1). It then outlines how data were collected using interviews and an online survey before giving details of the model development, parameterisation and the primary outcome.

### Respiratory panels

The two high-throughput respiratory PCR assays [13] can be run separately or simultaneously. They detect 19 different viral, bacterial and fungal pathogens commonly diagnosed in CAP patients, listed in Table 1. Respiratory 1 Panel (referred to here as Panel 1) detects pathogens associated with upper respiratory tract infections (10 viruses and 1 bacteria) and Respiratory 3 Panel (referred to here as Panel 3) detects pathogens associated with lower respiratory tract infections (8 bacteria and 1 fungus). The assays are semi-automated and provide results in 5.5 h. Both panels are approved for sale in the European Union (EU). Data on test performance were not yet available, it was assumed that the panels have diagnostic sensitivity and specificity comparable to PCR diagnostic tests that they would replace.

### Initial interviews with UK based clinicians and senior microbiologist

Four virtual semi-structured interviews were conducted to understand hospital care pathways, treatment strategies, and the potential value of the respiratory panels for patients with respiratory symptoms, including those admitted with CAP. Participants were a senior hospital microbiologist and three clinicians specialising in acute medicine, infectious diseases, and emergency paediatric medicine, all based at different hospitals in the UK and recruited through existing contacts. The interview data were used to inform which patient group to focus on, the development of the model and the online survey used to collect data for the model. One of the clinicians and the senior microbiologist also advised on which diagnostic tests currently used could be omitted if the respiratory panels were used.

### Survey data collection

A 10-min online survey was developed and used to collect data on clinical practice and the cost of diagnostic testing at present. The recruitment, translation (from English into French and Spanish) and collection of survey data was performed by an agency specialising in healthcare survey recruitment in Europe with a target sample size of 15 from each country. Data were collected during December 2021, in the UK, and January 2022, in France and Spain.

Senior healthcare professionals were invited to complete the survey. Participants had to be working in a hospital in the UK, France or Spain with 1) knowledge of which diagnostic tests are performed on adult patients admitted with CAP and 2) some understanding of the cost of these diagnostics to the hospital (both criteria being self-reported). Upon completion of the survey, participants received an honorarium for their time.

The data collected included the diagnostic tests used for adult patients admitted to hospital with CAP and the proportion of patients tested, the number of patients admitted with CAP and the turnaround time and cost of diagnostics, plus demographic information about the hospital including the geographic region, type of hospital, and private/public care offered. No patient identifying information was collected. The survey listed diagnostic tests that might be performed, based on the tests listed in the UK SMI recommendations for suspected CAP in immunocompetent and immunocompromised patients [14]. The survey allowed additional diagnostic assays not included in the SMI list to be listed.

The raw survey data were received in February 2022. The data were cleaned and analysed using Microsoft Excel 2022 (Microsoft Corporation, Redmond, USA). No statistical analysis was performed due to the small sample size. Median values for the percentage of patients tested and the cost of each test were used since data were not normally distributed.

### Model type and inputs

A cost-comparison model was developed to compare three testing strategies, the use of Panel 1, Panel 3 or Panel 1 plus Panel 3 versus standard of care (SoC).

The theoretical model included two key input parameters, 1) the percentage of patients tested with each diagnostic assay and 2) the median cost of each assay, calculated using the cost data reported in the survey. Data for each country (UK, France and Spain) and for immunocompromised and immunocompetent patients were analysed and reported separately due to the differences in clinical management.

### Primary outcome

The primary outcome assessed was the average cost of diagnostics per patient admitted with CAP. This was calculated using the median cost for each diagnostic test weighted by the percentage of patients that would have each test. For the panel strategies, this included the cost of the panel test (not necessarily for all patients, but for the proportion of patients who in SoC would otherwise have had diagnostic tests that could be replaced by the panel/s) plus the cost of diagnostics which would also be used, in addition to the respiratory panel (i.e., laboratory diagnostic tests not replaced by the panel).

For the panel strategies, the percentage of patients who would be tested using the respiratory panel was equal to the highest proportion of patients who would be tested in SoC with any of the diagnostic assays that could be replaced by the respiratory panel. For example, if Panel 1 could replace Assay A and Assay B and in SoC, 50% of patients have Assay A and 80% have Assay B, then in the Panel 1 strategy, 80% of patients would have Panel 1, 0% would have Assay A and 0% Assay B. The assumption being that all patients that would have been tested with Assay A are included in the group who would have Assay B.

### Time horizon

The time horizon considered was one episode of care i.e., one hospital admission, therefore no discounting of future costs was required.

### Costs

Costs were considered from a health systems perspective and presented in 2021–2022 UK British Pounds (£) and Euros (€) for the UK and Euros (€) for France and Spain. Costs were converted using an online cost convertor (https://www.xe.com/Currencyconverter), the conversion rate used was £1 to €1.19385. All the reported costs for diagnostic tests were assumed to include the cost of reagents/test kit plus laboratory staff time. The cost per patient per panel used in the model was €23.88 (£20.00) with -10% and + 20% used for the low and high values respectively. This estimated cost would include the reagents/test kit plus laboratory staff time.

The cost of tests that would be used to assess the patient but not used to diagnose the causative pathogen were not included in the model, for example, chest X-ray, pulse oximetry, respiratory rate, blood investigations, and diagnostic testing for non-respiratory pathogens. The cost of training clinical or laboratory staff or any required changes to laboratory procedures and equipment were not included.

### Deterministic sensitivity analysis (DSA)

In a one-way deterministic sensitivity analysis (DSA) performed for each country and each patient group (immunocompromised and immunocompetent), low and high values for each test were used to assess which parameters had the most effect on the average cost of diagnostics per patient. The results of the DSA were presented as a series of tornado plots which include the 10 most impactful variables.

For the DSA, for the respiratory panel strategies, 100% was used as the high value for the percentage of patients having the respiratory panel and 50% of the median was used for the low value.

### Scenario analyses

The median cost per hospital for diagnostic testing of patients admitted with CAP was calculated as well as the potential savings if Panel 1 + 3 strategy were used. These were calculated using the average number of patients with CAP admitted to a hospital per month, accounting for the ratio of immunocompromised and immunocompetent patients, multiplied by the median cost per patient.

To assess the average cost per patient if all CAP patients were tested with both respiratory panels, the average cost per patient admitted with CAP was calculated for the Panel 1 + 3 strategy where the baseline (median) percentage of patients having each of the diagnostic tests was used but all 100% patients had a sample tested using the respiratory panel/s.

### Patient and public involvement

There was no patient or public involvement in the design, conduct, reporting or dissemination plans of this research.

## Results

The descriptive data from the survey are reported first, including which diagnostic tests are used in patients admitted to hospital with CAP and which could be omitted when using the respiratory panels, followed by the results of the model, sensitivity and scenario analysis.

### Respondent characteristics

In total, 48 surveys were completed, 17 from the UK, 15 from France, and 16 from Spain. Survey respondents were from a variety of medical specialities including emergency medicine, pulmonology, intensive care and internal medicine, working at publicly funded general or university hospitals. There was a variety of geographic regions represented in each country. Data about the survey respondents and hospitals represented are presented in Supplementary Table 1.

### Diagnostic tests used in CAP patients

There are up to 37 different diagnostic tests used for patients admitted with CAP; 20 for immunocompetent patients and 37 for immunocompromised patients (Table 2). Respiratory 1 Panel could be used instead of 4 tests considered for immunocompetent patients and 5 tests considered for immunocompromised patients. Respiratory 3 Panel could be used in place of 7 tests considered for immunocompetent patients and 11 for immunocompromised patients. Since both panels test for *Mycoplasma pneumoniae*, if used simultaneously, the panels could be used instead of 9 tests considered in immunocompetent patients and 14 in immunocompromised patients (Table 2). No additional tests for respiratory pathogens, beyond those listed in the survey, were reported, although some respondents reported testing CAP patients for other (non-respiratory) pathogens such as human immunodeficiency virus (HIV), which were not included in the model.

**Table 2:** Diagnostic tests used for patients adm﻿itted with CAP in the UK, France and Spain

**Diagnostic test**	**Diagnostic tests considered categorised by patient immune status**		**Could this diagnostic test be omitted when the panels are used?**			**Median reported test turnaround time (hours)** ^a^		
	**Immunocompetent**	**Immunocompromised**	**Panel 1**	**Panel 3**	**Panel 1 + 3**	**UK**	**France**	**Spain**
Adenovirus screen	N	Y	Y	N	Y	24	8	48
Aspergillus serum antigen	N	Y	N	N	N	36.5	16.5	72
BAL culture	Y	Y	N	N	N	48	12	72
Blood culture	Y	Y	N	N	N	48	24	72
Chlamydophila sp serology	Y	Y	N	N	N	48	10	72
Chlamydophila sp PCR (respiratory sample)	Y	Y	N	N	N	36	8	12
COVID-19 PCR	Y	Y	Y	N	Y	4.5	5	12
Cryptococcus serum antigen	N	Y	N	N	N	48	7.5	12
Cytomegalovirus PCR (BAL sample)	N	Y	N	N	N	48	8	8
Cytomegalovirus PCR serum	N	Y	N	N	N	48	8	8
Cytomegalovirus PCR (sputum sample)	N	Y	N	N	N	48	8	8
Epstein-Barr Virus screen	N	Y	N	N	N	48	7.5	48
Legionella culture (BAL sample)	Y	Y	N	N	N	48	9	24
Legionella culture (sputum sample)	Y	Y	N	N	N	48	9	24
Legionella PCR (BAL sample)	Y	Y	N	Y	Y	48	9	10
Legionella PCR (pleural fluid sample)	Y	Y	N	Y	Y	48	9	10
Legionella PCR (sputum sample)	Y	Y	N	Y	Y	48	9	10
Legionella urinary antigen test	Y	Y	N	Y	Y	24	7	4
Mycobacterium culture (BAL sample)	Y	Y	N	N	N	72	12	72
Mycobacterium culture (sputum sample)	Y	Y	N	N	N	72	12	72
Mycobacterium PCR	Y	Y	N	N	N	48	9	4
Mycology culture (BAL sample)	N	Y	N	N	N	48	12	72
Mycology culture (sputum sample)	N	Y	N	N	N	48	12	72
Mycology PCR (BAL sample)	N	Y	N	N	N	48	12	12
Mycology PCR (sputum sample)	N	Y	N	N	N	48	12	12
*Mycoplasma pneumoniae* serology	Y	Y	Y	Y	Y	48	12	48
*Mycoplasma pneumoniae* PCR (respiratory sample)	Y	Y	Y	Y	Y	48	8	10
Nocardia culture	N	Y	N	N	N	48	12	72
Non-tuberculous mycobacteria^b^ PCR (BAL sample)	N	Y	N	N	N	48	12	4
Pleural fluid culture	Y	Y	N	N	N	48	24	48
*Pneumocystis jirovecii* IF (BAL sample)	N	Y	N	Y	Y	36.5	10	72
*Pneumocystis jirovecii* IF (sputum sample)	N	Y	N	Y	Y	36.5	10	72
*Pneumocystis jirovecii* PCR (BAL sample)	N	Y	N	Y	Y	48	8	72
*Pneumocystis jirovecii* PCR (sputum sample)	N	Y	N	Y	Y	48	8	72
Respiratory virus PCR screen (respiratory sample)	Y	Y	Y	N	Y	36	8	48
Sputum culture	Y	Y	N	N	N	48	24	72
*Streptococcus pneumoniae* urinary antigen test	Y	Y	N	Y	Y	24	7	4

*Abbreviations*: *BAL* Bronchoalveolar lavage, *ES* Spain, *FR* France, *IF* Immunofluorescence, *N* No, *PCR* Polymerase chain reaction, *sp* Species, *TB* Tuberculosis, *UK* United Kingdom, *Y* Yes. Shaded areas are used to indicate where diagnostic tests could be omitted when using the panel/s

^a^Turnaround time refers to the time from when the sample is collected to the result being available to the clinician. Median turnaround time based on reported data

^b^The convention is to refer to these as non-tuberculous mycobacteria (NTM), although the culture process does not differentiate between *Mycobacterium tuberculosis* (MTB) and NTM

### Patient characteristics and management in the UK, France and Spain

Data on admissions, CAP severity and the ratio of immunocompetent and immunocompromised patients are presented in Table 3. In the UK, France and Spain, the majority of CAP admissions are immunocompetent patients (72%, 55% and 67% respectively). In all three countries, most immunocompetent patients admitted with CAP have a CURB-65 score of 2 or above (82%, 72% and 77% in the UK, France and Spain respectively). The average number of adults admitted with CAP in an ‘average’ or a ‘busy’ month was *n* = 85 and *n* = 141 respectively in the UK hospitals, *n* = 42 and *n* = 69 in the French hospitals and *n* = 61 and *n* = 126 in the Spanish hospitals.

**Table 3:** Characteristics of patients at the hospitals included in an online survey, including number admitted per month, immune status and symptom severity of patients admitted with community-acquired pneumonia (CAP)

	**UK** **(** ***N*** ** = 16)**	**France** **(** ***N*** ** = 15)**	**Spain** **(** ***N*** ** = 15)**
**Mean number of adult CAP patients admitted, n (SD)**			
In a ‘typical’ month	85.3 (67.4)	41.7 (30.7)	60.8 (46.6)
In a ‘busy’ month	140.9 (101.7)	68.9 (49.6)	126.1 (80.6)
**Mean percentage of CAP admittances by immune status, % (SD)**			
Immunocompetent	76.3% (17.7%)	55.0% (22.8%)	67.0% (18.1%)
Immunocompromised	23.8%	45.0%	33.0%
**Mean percentage of admitted CAP patients by CURB-65** [11]** category, %**			
CURB-65 Score 0–1	17.9%	28.2%	22.8%
CURB-65 Score 2	40.2%	33.7%	43.9%
CURB-65 Score 3–5	41.9%	38.1%	33.3%

*Abbreviations*: *CAP* Community-acquired pneumonia, *CURB-65* New confusion, blood urea nitrogen (> 7 mmol/L), respiratory rate ≥ 30 breaths per minute, systolic blood pressure < 90 mmHg or diastolic blood pressure ≤ 60 mmHg, age ≥ 65 years, Pneumonia Severity Assessment [11], *n* number; N denominator, *SD* standard deviation

The proportion of patients tested using each diagnostic test varied widely between hospitals and between countries (details provided in Supplementary Tables 2–5 UK, Tables 6–9 France and Tables 10–13 Spain). The proportion of patients tested in SoC with each of the diagnostic assays, and the average cost of these are presented in Supplementary Tables 2–3 for the UK, Supplementary Tables 6–7 for France and Supplementary Tables 10–11 for Spain. The proportion of patients that would be tested with each of the diagnostic assays in the panel strategies are presented in Supplementary Tables 4–5 for the UK, Supplementary Tables 8–9 for France and Supplementary Tables 12–13 for Spain

### Average cost of diagnostic testing per patient admitted with CAP in SoC

In SoC, the average cost of diagnostic testing per immunocompetent patient was €85.99 (£72.03) in the UK, €277.58 in France and €127.85 in Spain. For immunocompromised patients, it was €102.34 (£85.73) in the UK, €379.96 in France and €211.11 in Spain (Table 4). The average cost was highest in France due to more testing and higher prices for diagnostic tests. For example, in France, of the 20 assays considered for immunocompetent patients, 17 were used in at least 25% of patients admitted with CAP, with each test costing €32.32 on average (Supplementary Table 6). In the UK, only 4 of the 20 tests were used in at least 25% of immunocompetent patients with each costing £20.00 (€23.88) on average (Supplementary Table 2) and in Spain, 6 of the 20 tests were used in at least 25% of immunocompetent patients at an average cost of (€20.00) per test (Supplementary Table 10).

**Table 4:** Average cost of diagnostic testing per patient admitted with CAP in the UK, France, and Spain

Strategy	UK			France			Spain		
	**Base case**	**Low**	**High** ^b^	**Base case**	**Low**	**High** ^b^	**Base case**	**Low**	**High** ^b^
Immunocompetent patients									
SoC	€ 85.99	€ 1.49	€ 695.95	€ 277.58	€ 5.40	€ 1,656.65	€ 127.85	€2.59	€ 1,460.85
Panel 1^a^	€ 66.89	€ 12.24	€ 488.27	€ 245.75	€ 15.14	€ 1,265.30	€ 102.98	€ 12.85	€ 1,047.82
Panel 3	€ 81.81	€ 3.64	€ 467.80	€ 197.10	€ 15.24	€ 995.30	€ 120.40	€ 10.79	€ 1,007.07
Panel 1 + 3	€ 63.90	€ 14.39	€ 329.31	€ 177.98	€ 24.99	€ 803.95	€ 100.78	€ 21.05	€ 747.39
Difference between SoC and Panel 1 + 3^c^	-€ 22.09	€ 12.89	-€ 366.64	-€ 99.60	€ 19.59	-€ 852.70	-€ 27.07	€ 18.46	-€ 713.46
Immunocompromised patients									
SoC	€ 102.34	€ 1.79	€ 1,228.93	€ 379.96	€ 8.25	€ 2,931.50	€ 211.11	€8.80	€ 3,063.62
Panel 1^a^	€ 87.42	€ 12.54	€ 982.75	€ 349.73	€ 16.99	€ 2,430.15	€ 177.55	€ 16.17	€ 2,558.24
Panel 3	€ 88.17	€ 5.01	€ 800.17	€ 292.81	€ 16.85	€ 1,870.15	€ 188.36	€ 17.46	€ 2,023.17
Panel 1 + 3	€ 76.23	€ 15.76	€ 636.61	€ 271.18	€ 25.59	€ 1,568.80	€ 159.24	€ 25.08	€ 1,738.49
Difference between SoC and Panel 1 + 3^c^	-€ 26.12	€ 13.97	-€ 592.32	-€ 108.77	€ 17.34	-€ 1,362.70	-€ 51.87	€ 16.28	-€ 1,325.13

*Abbreviations*: *SoC* Standard of care, *UK* United Kingdom. Equivalent UK costs in UK £ are presented in Supplementary Table 14

^a^Panel 1 strategy refers to the cost of using Respiratory 1 Panel to replace some SoC tests plus the cost of diagnostic tests not replaced by the panel. The equivalent is true for the Panel 3 strategy and for the Panel 1 + 3 strategy

^b^For respiratory panel strategies, the high value refers to 100% of people being tested with the relevant respiratory panel/s

^c^A negative number here indicates that the Panel 1 + 3 strategy results in cost savings compared to SoC

### Average cost of diagnostic testing per patient admitted with CAP using the respiratory panels

In all three countries and for immunocompetent and immunocompromised patients, all three respiratory panel strategies cost less than SoC (Table 4) with the Panel 1 + 3 strategy provided the biggest cost saving. Compared to SoC, the Panel 1 + 3 strategy reduced the average cost of diagnostics per immunocompetent patient by €22.09 (£18.50) in the UK, €99.60 in France and €27.07 in Spain. Cost savings were greater in immunocompromised patients, with the Panel 1 + 3 strategy reducing the average cost of diagnostics compared to SoC by €26.12 (£21.88) in the UK, €108.77 in France and €51.87 in Spain. When using the high values, the savings from Panel 1 + 3 strategy compared to SoC were higher (Table 4). When using the low values, the Panel 1 + 3 strategy cost more than SoC.

### One way sensitivity analysis

In the DSA, in SoC the cost of the respiratory virus PCR screen and the cost of the COVID-19 PCRs were among the variables with the largest effect on the average cost per patient for immunocompetent and immunocompromised patients in all three countries (Supplementary Fig. 1–3 A + B). In France, the cost of legionella urinary antigen tests also had a large effect (Supplementary Fig. 2 A + B) as did the cost of *S. pneumoniae* urinary antigen testing in Spain (Supplementary Fig. 3 A + B).

For the Panel 1 + 3 strategy, the cost of blood cultures was the most impactful variable for all patient groups in all three countries (Supplementary Fig. 1–3 C + D) with the exception of immunocompetent patients in Spain, where the cost of COVID-19 PCR had the biggest impact (Supplementary Fig. 3C).

### Scenario analysis

The total cost of diagnostic testing for immunocompromised and immunocompetent patients admitted with CAP was calculated for each country for SoC and the Panel 1 + 3 strategy for ‘typical’ months and ‘busy’ months (Supplementary Table 15). In UK hospitals, the average cost per hospital on ‘typical’ months was €7,666 (£6,421) in SoC compared to €5,700 (£4,775) for the Panel 1 + 3 strategy, resulting in a potential saving of €1,966 (£1,646) per month. For hospitals in France, the cost savings per month were €4,325 (SoC: €13,496 *vs*. Panel 1 + 3: €9,171) and in Spain, €2,144 (SoC: €9,444 *vs*. Panel 1 + 3: €7,300). On ‘busy’ months the savings from using Panel 1 + 3 strategy compared to SoC were higher (€3,247 [£2,716] in the UK, €7,147 in France and €4,446 in Spain).

For the base case model, 100% of patients had testing with Respiratory 1 Panel in the Panel 1 strategy and the Panel 1 + 3 strategy because one of the tests this panel replaces is the COVID-19 PCR, and 100% of patients had the COVID-19 PCT in SoC in all three countries (Supplementary Tables 2–3, 6–7 and 10–11). The percentage of patients tested with Respiratory 3 Panel in the base case for immunocompetent and immunocompromised patients respectively was 20% and 30% in the UK, 100% and 80% in France and 80% and 95% in Spain. This value was based on the percentage of patients being tested for either Legionella or *S. pneumoniae*, as the percentage of patients tested with these was higher than for the other tests replaced by Respiratory 3 Panel.

In scenario analysis, when 100% of patients received testing with both panels for the panel strategies, the average cost per patient for the Panel 1 + 3 strategy was less than for SoC (Supplementary Table 16). The savings per patient for immunocompetent and immunocompromised patients respectively were €2.98 (£2.50) and €9.40 (£7.88) in the UK, €99.60 and €104.00 in France and €22.30 and €50.68 in Spain.

## Discussion

### Key findings

If the key assumptions of the model are correct, the findings from this cost comparison indicate that with the introduction of these two respiratory panels, hospitals in England, France and Spain could save €2,000 to €7,000 each month per hospital by replacing some of the diagnostic tests currently used to assess patients admitted with CAP. If all patients admitted with CAP, whether immunocompromised or immunocompetent, were tested using both panels replacing some of the diagnostic tests currently used, there could be considerable savings to the health system in all three countries since such large numbers of patients are admitted with CAP.

Since the early months of the COVID pandemic, hospitals have been testing many patients for COVID-19, not only patients presenting with respiratory symptoms. We anticipate that COVID-19 testing practice will change somewhat in the future, as will the funding structure for these tests, but that there would continue to be testing for COVID-19 in patients admitted with respiratory symptoms and as such, the cost calculations would remain valid even as COVID-19 testing within the wider health system in each country changes.

### Strengths of the study

This study collected information from senior clinicians on current practice regarding the proportion of CAP patients tested for different pathogens and the cost to the health system of these tests, representing a wide geographical spread in three countries. This provides a framework for assessing the utility of respiratory panels for the identification of pathogenic agents in CAP patients on the types of diagnostic tests performed and the percentage of patients being tested and provides useful data, particularly on the cost of diagnostics, not typically available in published literature.

There was considerable variation between countries and between hospitals, in the cost of diagnostics and in the percentage of patients tested for different pathogens. This is likely to reflect differences in clinical practice as well as differences in the severity of symptoms and presentation of patients being admitted. The sample size was relatively small, and the hospitals included in the survey may not be representative of all hospitals within each country. However, in finding that the Panel 1 + 3 strategy provided savings in each of the three countries, where the levels of testing and the cost of diagnostics differ, and for immunocompetent and immunocompromised patient groups separately, this gives some confidence that cost savings are likely in a wide variety of locations.

### Limitations

This study used a survey methodology to collect data from a range of clinicians. However, as observed with survey methodologies, there are risks with inconsistent and incorrect responses. We minimised this risk by collecting data from senior consultants and translating the survey into French and Spanish for clinicians in France and Spain. However, some turnaround times reported in the survey did not appear to be accurate. For example, a turnaround time of less than 12-h for some culture assays and it was not possible to query the reported results retrospectively. It was decided not to exclude these data since although they were included in the results section, they were not used to inform the main analysis. In future, data quality could be improved by complementing online surveys with clinician interviews, particularly in France and Spain where no interviews were carried out.

It is difficult to test the validity of the cost data. The cost per assay is not typically reported by laboratories since these are not standardised costs and vary according to the quantity purchased as well as what additional assays, equipment or laboratory materials are purchased. To minimise the inclusion of any extreme values, the median value was used. To improve reliability, in future similar studies, a separate survey to collect cost data could be targeted at senior microbiologist.

When developing this model, data on the sensitivity and specificity of the panels to detect pathogens was not available nor on how they compare with the diagnostic tests currently used – therefore it was assumed that the panels would have equivalent performance to the comparable molecular tests that they would replace. Evidence on the clinical performance of the panel test, once available, should be used alongside this cost related evidence to support decision making as to whether a laboratory chooses to swap out existing assays with the panel tests.

An important assumption made in the model was that the proportion of people who would have a sample tested using the respiratory panel/s in the panel strategies was equal to the highest proportion who would receive any one of the tests that the panel could replace. This means that the proportion of people who would have the panel could be underestimated, thereby underestimating the cost of the panel strategies. However, even when, in scenario analysis, all patients were tested with both respiratory panels, the Panel 1 + 3 strategy still cost less than SoC.

The cost to purchase and install new laboratory equipment and train laboratory staff and clinicians in new procedures was not considered. High throughput PCR machines are standard equipment in modern microbiology laboratories and training could be incorporated into ongoing professional development.

### Context

Previous studies have reported cost savings from optimising the antimicrobial prescribing decisions and shorter hospital admissions with the use of diagnostics which provide a swifter result for patients (adults and children) presenting with respiratory style infections including CAP, the common cold, otitis media and others [15, 16]. In many cases but not all, the respiratory panels would provide a quicker result than the test/s they replace. By considering only the average cost of diagnostics, our model did not compare the information available to clinicians in SoC versus the panel strategies or the timing of that information. On the one hand, the respiratory panels could provide more information to guide patient management compared to SoC, since some tests included in the panel might not be run routinely in SoC or only performed once preliminary tests were negative. Previous studies suggest that using a respiratory panel in patients admitted with CAP increases the proportion with a potential pathogen identified compared with a standard multi-test diagnostic bundle [15]. On the other hand, where the panel/s replaces more time-consuming standard diagnostic tests such as culture, it provides less information, since a PCR test gives a binary result (positive or negative) rather than a quantitative result. In some cases, culture may still be required for antibiotic susceptibility testing.

### Future work

For most tests, the turnaround time from using the respiratory panel/s was faster than the turnaround time would be for diagnostic tests used in SoC (Table 2). A notable exception was for COVID-19 which had a median turnaround time of 4.5 h in the UK hospitals and 5 h in France (12 h in Spain). Rapid tests with ≤ 30-min turnaround are widely used for COVID-19, and it was assumed that patients being admitted with CAP will have already been tested using a rapid test on arrival at hospital. For other pathogens, a quicker result from using the respiratory panels could impact clinical management and patient outcomes, including reducing the duration of hospital stay and duration on unnecessary/suboptimal antibiotics. Therefore, assessing only the costs associated with diagnostics is likely to underestimate the potential cost-effectiveness of the respiratory panels. To assess the full extent to which the use of the respiratory panels would impact clinical outcomes and other costs related to antibiotic use and hospital admission requires a full clinical evaluation, data from which could be used to inform a more complex cost-effectiveness model. The impact of changing from existing molecular diagnostics to a panel test on the operational aspects of laboratory processes and workflow were not considered in the model and would also require a real-world evaluation to assess.

After a discussion with a paediatric specialist, this study was restricted to focus on diagnostic testing in adults, since the majority of children are not diagnosed with a cause of CAP and are typically symptomatically managed. There would be value in exploring which diagnostic tests are used and the percentage of children being tested with each since approximately 14.4 per 10,000 children aged over 5 years and 33.8 per 10,000 under 5 years are diagnosed with CAP annually in European hospitals [17].

CAP was the only indication considered in the model. However, the respiratory panels may well be of value for other types of patients including those with suspected respiratory presentations such as healthcare-associated pneumonia (HAP) which, in addition to CAP, has a significant burden of disease in Europe [18]. Inappropriate antibiotic prescribing in upper respiratory tract infections is a common issue observed globally in primary care and represents a significant number of attendances to hospital for infectious disease review [19, 20]. Use of respiratory panels in the diagnostic screening of upper respiratory tract infections could help target treatment more quickly and have direct implications for antimicrobial stewardship in avoiding the use of antibiotics in patients who present with viral infections. This area should be explored further.

## Conclusions

This preliminary costing work suggests that, although there is variation in current practice related to diagnostic testing of adults admitted to hospital with CAP, replacing current tests with these high-throughput laboratory-based respiratory PCR panels could save costs in high-income European countries.

### Supplementary Information

**Additional file 1:** **Supplementary Table 1. **Descriptive data from online survey for the UK, France and Spain. **Supplementary Table 2. **Diagnostic tests used for immunocompetent patients admitted with CAP (SoC) in the UK. **Supplementary Table 3.** Diagnostic tests used for immunocompromised patients admitted with CAP (SoC) in the UK. **Supplementary Table 4. **Diagnostic tests used for immunocompetent patients admitted with CAP (panel test strategies) in the UK. **Supplementary Table 5. **Diagnostic tests used for immunocompromised patients admitted with CAP (panel test strategies) in the UK. **Supplementary Table 6. **Diagnostic tests used for immunocompetent patients admitted with CAP (SoC) in France. **Supplementary Table 7. **Diagnostic tests used for immunocompromised patients admitted with CAP (SoC) in France. **Supplementary Table 8. **Diagnostic tests used for immunocompetent patients admitted with CAP (panel test strategies) in France. **Supplementary Table 9. **Diagnostic tests used for immunocompromised patients admitted with CAP (panel test strategies) in France. **Supplementary Table 10.** Diagnostic tests used for immunocompetent patients admitted with CAP (SoC) in Spain. **Supplementary Table 11. **Diagnostic tests used for immunocompromised patients admitted with CAP (SoC) in Spain. **Supplementary Table 12. **Diagnostic tests used for immunocompetent patients admitted with CAP (panel test strategies) in Spain. **Supplementary Table 13. **Diagnostic tests used for immunocompromised patients admitted with CAP (panel test strategies) in Spain. **Supplementary Table 14. **Average cost of diagnostic testing per immunocompetent and immunocompromised patient admitted with CAP in the UK. **Supplementary Table 15. **Estimated monthly costs per hospital of diagnostic testing of patients admitted with CAP. **Supplementary Table 16. **Average cost per patient admitted with CAP if 100% were tested with both panel tests compared to SoC. **Supplementary Figure 1. **UK: Tornado plots showing impact of key parameters on average cost per patient for SoC and Panel 1+3 strategy. **Supplementary Figure 2. **France: Tornado plots showing impact of key parameters on average cost per patient for SoC and Panel 1+3 strategy. **Supplementary Figure 3. **Spain: Tornado plots showing impact of key parameters on average cost per patient for SoC and Panel 1+3 strategy. 

## Data Availability

All data relevant to the study are included in the article or uploaded as supplementary material.
