# Correction to: an economic evaluation of two PCR-based respiratory panel assays for patients admitted to hospital with community-acquired pneumonia (CAP) in the UK, France and Spain

**DOI:** 10.1186/s12890-023-02572-8

**Published:** 2023-08-11

**Authors:** Lisa Miners, Susie Huntington, Nathaniel Lee, Katy M. E. Turner, Elisabeth Adams

**Affiliations:** 1 Aquarius Population Health, Unit 29 Tileyard Studios, N7 9AH London, UK; 2 Hospital for Tropical Diseases, University College London Hospital NHS Foundation Trust, London, UK

**BMC Pulmonary Medicine (2023) 23:220**

10.1186/s12890-023-02516-2

Following publication of the original article, it was brought to the authors’ attention that the names of collaborators had been omitted from the acknowledgements and that the funding details were incorrect.

The details of the collaborators added to the acknowledgements are as follows:

We thank the following collaborators for their contributions: Fernando Fernandez (Head of Microbiology Unit), Francisco Rivas (Coordinator of Methodology Unit) and Marilina Garcia (RAPID-COVID Postdoctoral Researcher) all from Hospital Costa del Sol, Marbella, Spain, and Martin Rottman from Faculté de Médecine Simone Veil, Université de Versailles St Quentin, France and Innovative Biomarkers Platform, Hôpital Raymond Poincaré, France.

The details of the funding have been changed and the updated text now reads:

This project received funding from the Innovative Medicines Initiative 2 Joint Undertaking (JU) under grant agreement No 101005144 awarded to Genefirst and collaborating organisations. The JU receives support from the European Union’s Horizon 2020 research and innovation programme and EFPIA.

These errors have since been corrected in the original article. The authors apologize for any inconvenience caused.

