# Supplementary material for: An economic evaluation of two PCR-based respiratory panel assays for patients admitted to hospital with community-acquired pneumonia (CAP) in the UK, France and Spain
Source: BMC Pulm Med. 2023 Jun 21;23:220. doi: 10.1186/s12890-023-02516-2 (PMC10283259; doi:10.1186/s12890-023-02516-2)
Supplement: Supplementary file 1 — Additional file 1: Supplementary Table 1. Descriptive data from online survey for the UK, France and Spain. Supplementary Table 2. Diagnostic tests used for immunocompetent patients admitted with CAP (SoC) in the UK. Supplementary Table 3. Diagnostic tests used for immunocompromised patients admitted with CAP (SoC) in the UK. Supplementary Table 4. Diagnostic tests used for immunocompetent patients admitted with CAP (panel test strategies) in the UK. Supplementary Table 5. Diagnostic tests used for immunocompromised patients admitted with CAP (panel test strategies) in the UK. Supplementary Table 6. Diagnostic tests used for immunocompetent patients admitted with CAP (SoC) in France. Supplementary Table 7. Diagnostic tests used for immunocompromised patients admitted with CAP (SoC) in France. Supplementary Table 8. Diagnostic tests used for immunocompetent patients admitted with CAP (panel test strategies) in France. Supplementary Table 9. Diagnostic tests used for immunocompromised patients admitted with CAP (panel test strategies) in France. Supplementary Table 10. Diagnostic tests used for immunocompetent patients admitted with CAP (SoC) in Spain. Supplementary Table 11. Diagnostic tests used for immunocompromised patients admitted with CAP (SoC) in Spain. Supplementary Table 12. Diagnostic tests used for immunocompetent patients admitted with CAP (panel test strategies) in Spain. Supplementary Table 13. Diagnostic tests used for immunocompromised patients admitted with CAP (panel test strategies) in Spain. Supplementary Table 14. Average cost of diagnostic testing per immunocompetent and immunocompromised patient admitted with CAP in the UK. Supplementary Table 15. Estimated monthly costs per hospital of diagnostic testing of patients admitted with CAP. Supplementary Table 16. Average cost per patient admitted with CAP if 100% were tested with both panel tests compared to SoC. Supplementary Figure 1. UK: Tornado plots showing impact of key parameters on average [file 12890_2023_2516_MOESM1_ESM.docx]

**Supplementary Material: An economic evaluation of two PCR-based respiratory panel assays for patients admitted to hospital with community-acquired pneumonia (CAP) in the UK, France and Spain**

**Contents**

| Supplementary Table 1. | Descriptive data from online survey for the UK (n=17), France (n=15) and Spain (n=16) | Page 3 |
| --- | --- | --- |
|  |  |  |
| **UK** |  |  |
| Supplementary Table 2. | Diagnostic tests used for immunocompetent patients admitted with CAP (SoC) | Page 5 |
| Supplementary Table 3. | Diagnostic tests used for immunocompromised patients admitted with CAP (SoC) | Page 6 |
| Supplementary Table 4. | Diagnostic tests used for immunocompetent patients admitted with CAP (Panel 1, Panel 3 and Panel 1+3) | Page 8 |
| Supplementary Table 5. | Diagnostic tests used for immunocompromised patients admitted with CAP (Panel 1, Panel 3 and Panel 1+3) | Page 9 |
|  |  |  |
| **France** |  |  |
| Supplementary Table 6. | Diagnostic tests used for immunocompetent patients admitted with CAP (SoC) | Page 11 |
| Supplementary Table 7. | Diagnostic tests used for immunocompromised patients admitted with CAP (SoC) | Page 12 |
| Supplementary Table 8. | Diagnostic tests used for immunocompetent patients admitted with CAP (Panel 1, Panel 3 and Panel 1+3) | Page 14 |
| Supplementary Table 9. | Diagnostic tests used for immunocompromised patients admitted with CAP (Panel 1, Panel 3 and Panel 1+3) | Page 15 |
|  |  |  |
| **Spain** |  |  |
| Supplementary Table 10. | Diagnostic tests used for immunocompetent patients admitted with CAP (SoC) | Page 17 |
| Supplementary Table 11. | Diagnostic tests used for immunocompromised patients admitted with CAP (SoC) | Page 18 |
| Supplementary Table 12. | Diagnostic tests used for immunocompetent patients admitted with CAP (Panel 1, Panel 3 and Panel 1+3) | Page 20 |
| Supplementary Table 13. | Diagnostic tests used for immunocompromised patients admitted with CAP (Panel 1, Panel 3 and Panel 1+3) | Page 21 |
|  |  |  |
| Supplementary Table 14. | Average cost of diagnostic testing per immunocompetent and immunocompromised patient admitted with CAP in UK (£) | Page 23 |
| Supplementary Table 15. | Scenario analysis: Estimated monthly costs per hospital | Page 24 |
| Supplementary Table 16. | Average cost per patient admitted with CAP if 100% were tested with both panel tests compared to SoC | Page 25 |
|  |  |  |
|  |  |  |
| **Sensitivity Analysis** |  |  |
| Supplementary Figure 1 | UK: Tornado plots showing impact of key parameters on average cost per patient for SoC and Panel 1+3 strategy | Page 26 |
| Supplementary Figure 2 | France: Tornado plots showing impact of key parameters on average cost per patient for SoC and Panel 1+3 strategy | Page 27 |
| Supplementary Figure 3 | Spain: Tornado plots showing impact of key parameters on average cost per patient for SoC and Panel 1+3 strategy | Page 28 |

**Supplementary Table 1. Descriptive data from online survey for the UK (n=17), France (n=15) and Spain (n=16)**

| **UK** | **n (%)** | **France** | **n (%)** | **Spain** | **n (%)** |
| --- | --- | --- | --- | --- | --- |
| **Medical Specialty** |  |  |  |  |  |
| Consultant | 4 (23.5) | Consultant | 4 (26. 7) | Consultant | 2 (12.5) |
| Emergency Medicine | 4 (23.5) | Infectious Diseases | 1 (6.7) | Emergency Medicine | 1 (6.3) |
| Microbiologist | 1 (5.9) | Intensive care | 1 (6.7) | Infectious Diseases | 3 (18.8) |
| Physician/ Practitioner | 1 (5.9) | Pharmacist | 1 (6.7) | Internal medicine | 2 (12.5) |
| Respiratory | 7 (41.2) | Physician/ Practitioner | 7 (46.7) | Physician/ Practitioner | 1 (6.3) |
|  |  | Pulmonologist | 1 (6.7) | Pulmonologist | 6 (37.5) |
|  |  |  |  | Hospital researcher | 1 (6.3) |
| **Geographical region** | | | | | |
| England, London | 1 (5.9) | Auvergne-Rhône-Alpes | 2 (13.3) | Andalusia | 2 (12.5) |
| England, Midlands | 2 (11.8) | Ile de France (Paris) | 4 (26.7) | Castile and León | 1 (6.3) |
| England, Northwest | 3 (17.7) | Nouvelle-Aquitaine | 2 (13.3) | Castilla-La Mancha | 3 (18.8) |
| England, Southeast | 2 (11.8) | Occitanie | 3 (20.0) | Catalonia | 2 (12.5) |
| England, Southwest | 3 (17.7) | Provence-Côte d’Azur | 1 (6.7) | Extremadura | 1 (6.3) |
| England, Yorkshire | 1 (5.9) | Not reported | 3 (20.0) | Madrid | 5 (31.3) |
| Scotland | 2 (11.8) |  |  | Valencia | 1 (6.3) |
| Not reported | 3 (17.7) |  |  | Not reported | 1 (6.3) |
| **Hospital type** | | | | | |
| General Hospital | 6 (35.3) | General Hospital | 5 (33.3) | General Hospital | 2 (12.5) |
| University Hospital | 9 (52.9) | University Hospital | 10 (66.7) | University Hospital | 14 (87.5) |
| Not reported | 2 (11.8) |  |  |  |  |

Table 1 continued on the next page.

Table 1 continued from the previous page.

| **UK** | **n (%)** | **France** | **n (%)** | **Spain** | **n (%)** |
| --- | --- | --- | --- | --- | --- |
| **Hospital department where CAP patients would be admitted** | | | | | |
| Emergency Department | 5 (29.4) | Ear, Nose and Throat Medicine | 1 (6.7) | Emergency Department | 1 (6.3) |
| Internal Medicine | 1 (5.9) | Infectious Diseases | 1 (6.7) | Infectious Diseases | 2 (12.5) |
| Respiratory | 6 (35.3) | Intensive Care | 2 (13.3) | Internal Medicine | 1 (6.3) |
| Not reported | 5 (29.4) | Pneumology | 4 (26.6) | Intensive Care | 1 (6.3) |
|  |  | Not reported | 7 (46.7) | Pneumology | 10 (62.5) |
|  |  |  |  | Not reported | 1 (6.3) |

**Supplementary Table 2. Diagnostic tests used for immunocompetent patients admitted with CAP in the UK (SoC)**

| **Diagnostic test** | **CAP patients tested, %** | | | | | **Cost (per unit) to health service, £** | | | |
| --- | --- | --- | --- | --- | --- | --- | --- | --- | --- |
|  | **Median** | **Low value** | **High value** | **N** | **Median** | | **Low value** | **High value** | **N** |
| Respiratory 1 Panel | - | - | - | - | 20.00 | | 18.00 | 24.00 | - |
| Respiratory 3 Panel | - | - | - | - | 20.00 | | 18.00 | 24.00 | - |
| Pleural fluid culture | 5 | 0 | 30 | 16 | 26.50 | | 5.00 | 50.00 | 10 |
| Blood culture | 80 | 15 | 100 | 17 | 20.00 | | 5.00 | 40.00 | 13 |
| Sputum culture | 50 | 10 | 100 | 17 | 15.00 | | 5.00 | 29.00 | 12 |
| BAL culture | 5 | 0 | 20 | 17 | 25.00 | | 10.00 | 100.00 | 9 |
| COVID-19 PCR | 100 | 0 | 100 | 17 | 20.00 | | 5.00 | 50.00 | 15 |
| Respiratory virus PCR screen (respiratory sample) | 60 | 0 | 100 | 15 | 25.00 | | 5.00 | 90.00 | 11 |
| Legionella culture (sputum sample) | 1 | 0 | 90 | 17 | 20.00 | | 5.00 | 57.62 | 8 |
| Legionella PCR (sputum sample) | 0 | 0 | 40 | 17 | 20.00 | | 5.00 | 111.96 | 7 |
| Legionella culture (BAL sample) | 1 | 0 | 10 | 16 | 20.00 | | 5.00 | 57.62 | 8 |
| Legionella PCR (BAL sample) | 0 | 0 | 10 | 17 | 20.00 | | 5.00 | 111.96 | 7 |
| Legionella PCR (pleural fluid sample) | 0 | 0 | 10 | 17 | 20.00 | | 5.00 | 111.96 | 7 |
| Legionella urinary antigen test | 15 | 0 | 90 | 17 | 20.00 | | 5.00 | 54.97 | 10 |
| *Streptococcus pneumoniae* urinary antigen test | 20 | 0 | 90 | 17 | 17.50 | | 5.00 | 45.00 | 10 |
| Chlamydophila sp PCR (respiratory sample) | 1 | 0 | 15 | 17 | 20.00 | | 5.00 | 40.00 | 8 |
| Chlamydophila sp serology | 0 | 0 | 10 | 16 | 20.00 | | 5.00 | 25.00 | 6 |
| Mycobacterium culture (BAL sample) | 3 | 0 | 50 | 17 | 30.00 | | 5.00 | 43.72 | 9 |
| Mycobacterium culture (sputum sample) | 5 | 0 | 50 | 17 | 30.00 | | 5.00 | 43.72 | 9 |
| Mycobacterium PCR | 1.5 | 0 | 20 | 16 | 30.00 | | 5.00 | 70.00 | 6 |
| *Mycoplasma pneumoniae* PCR (respiratory sample) | 1 | 0 | 20 | 16 | 20.00 | | 5.00 | 115.92 | 9 |
| *Mycoplasma pneumoniae* serology | 4 | 0 | 30 | 16 | 20.00 | | 5.00 | 115.92 | 9 |

Abbreviations: BAL, bronchoalveolar lavage, CAP, community acquired pneumonia; COVID-19, SARS-CoV-2; N, the denominator used to inform the median, low and high values; PCR, polymerase chain reaction; SoC, standard of care; sp, species; UK, United Kingdom.

**Supplementary Table 3. Diagnostic tests used for immunocompromised patients admitted with CAP in the UK (SoC)**

| **Diagnostic test** | **CAP patients tested, %** | | | | | **Cost (per unit) to health service, £** | | | |
| --- | --- | --- | --- | --- | --- | --- | --- | --- | --- |
|  | **Median** | **Low value** | **High value** | **N** | **Median** | | **Low value** | **High value** | **N** |
| Blood culture | 80 | 20 | 100 | 17 | 20.00 | | 5.00 | 40.00 | 13 |
| Sputum culture | 40 | 10 | 95 | 17 | 15.00 | | 5.00 | 29.00 | 12 |
| BAL culture | 10 | 0 | 30 | 17 | 25.00 | | 10.00 | 100.00 | 9 |
| Pleural fluid culture | 5 | 0 | 20 | 16 | 26.50 | | 5.00 | 50.00 | 10 |
| COVID-19 PCR | 100 | 0 | 100 | 16 | 20.00 | | 5.00 | 50.00 | 15 |
| Respiratory virus PCR screen | 40 | 0 | 100 | 15 | 25.00 | | 5.00 | 90.00 | 11 |
| Legionella culture (sputum sample) | 0 | 0 | 90 | 16 | 20.00 | | 5.00 | 57.62 | 8 |
| Legionella PCR (sputum sample) | 0 | 0 | 50 | 16 | 20.00 | | 5.00 | 111.96 | 7 |
| Legionella culture (BAL sample) | 1 | 0 | 50 | 17 | 20.00 | | 5.00 | 57.62 | 8 |
| Legionella PCR (BAL sample) | 0 | 0 | 20 | 16 | 20.00 | | 5.00 | 111.96 | 7 |
| Legionella PCR (pleural fluid sample) | 0 | 0 | 20 | 16 | 20.00 | | 5.00 | 111.96 | 7 |
| Legionella urinary antigen test | 30 | 0 | 100 | 17 | 20.00 | | 5.00 | 54.97 | 10 |
| *Streptococcus pneumoniae* urinary antigen test | 30 | 0 | 100 | 17 | 17.50 | | 5.00 | 45.00 | 10 |
| Chlamydophila sp PCR (respiratory sample) | 1 | 0 | 20 | 16 | 20.00 | | 5.00 | 40.00 | 8 |
| Chlamydophila sp serology | 1 | 0 | 30 | 17 | 20.00 | | 5.00 | 25.00 | 6 |
| Mycobacterium culture (BAL sample) | 7.5 | 0 | 75 | 16 | 30.00 | | 5.00 | 43.72 | 9 |
| Mycobacterium culture (sputum sample) | 10 | 0 | 60 | 16 | 30.00 | | 5.00 | 43.72 | 9 |
| Mycobacterium PCR | 7.5 | 0 | 40 | 16 | 30.00 | | 5.00 | 70.00 | 6 |
| *Mycoplasma pneumoniae* PCR (respiratory sample) | 5 | 0 | 39 | 17 | 20.00 | | 5.00 | 115.92 | 9 |
| *Mycoplasma pneumoniae* serology | 7.5 | 0 | 50 | 16 | 20.00 | | 5.00 | 48.00 | 8 |
| Mycology culture (BAL sample) | 5 | 0 | 20 | 16 | 20.00 | | 5.00 | 40.00 | 7 |
| Mycology culture (sputum sample) | 1.5 | 0 | 50 | 16 | 20.00 | | 5.00 | 40.00 | 7 |
| Mycology PCR (BAL sample) | 1 | 0 | 40 | 16 | 30.00 | | 5.00 | 50.00 | 6 |
| Mycology PCR (sputum sample) | 0 | 0 | 39 | 16 | 30.00 | | 5.00 | 50.00 | 6 |

Table 3 continued on the next page.

Table 3 continued from the previous page.

| **Diagnostic test** | **CAP patients tested, %** | | | | | **Cost (per unit) to health service, £** | | | |
| --- | --- | --- | --- | --- | --- | --- | --- | --- | --- |
|  | **Median** | **Low value** | **High value** | **N** | **Median** | | **Low value** | **High value** | **N** |
| *Pneumocystis jirovecii* PCR (BAL sample) | 10 | 0 | 49 | 16 | 25.00 | | 5.00 | 80.00 | 7 |
| *Pneumocystis jirovecii* PCR (sputum sample) | 0.5 | 0 | 40 | 16 | 25.00 | | 5.00 | 80.00 | 7 |
| *Pneumocystis jirovecii* IF (BAL sample) | 5 | 0 | 40 | 17 | 25.00 | | 5.00 | 60.00 | 7 |
| *Pneumocystis jirovecii* IF (sputum sample) | 1 | 0 | 30 | 17 | 25.00 | | 5.00 | 60.00 | 7 |
| Adenovirus screen | 0 | 0 | 30 | 16 | 20.00 | | 5.00 | 70.00 | 9 |
| Aspergillus serum antigen | 3 | 0 | 40 | 16 | 12.50 | | 5.00 | 50.00 | 8 |
| Cryptococcus serum antigen | 1 | 0 | 30 | 17 | 20.00 | | 5.00 | 30.00 | 6 |
| Cytomegalovirus PCR (BAL sample) | 0 | 0 | 30 | 16 | 25.00 | | 5.00 | 60.00 | 8 |
| Cytomegalovirus PCR (sputum sample) | 0 | 0 | 30 | 16 | 25.00 | | 5.00 | 60.00 | 8 |
| Cytomegalovirus PCR serum | 0 | 0 | 30 | 16 | 25.00 | | 5.00 | 60.00 | 8 |
| Epstein-Barr Virus screen | 5 | 0 | 30 | 16 | 20.00 | | 5.00 | 50.00 | 8 |
| Nocardia culture | 0 | 0 | 10 | 16 | 30.00 | | 5.00 | 50.00 | 7 |
| Non-TB mycobacteria PCR (BAL sample) | 2.5 | 0 | 40 | 16 | 30.00 | | 5.00 | 60.00 | 7 |

Abbreviations: BAL, bronchoalveolar lavage, CAP, community acquired pneumonia; COVID-19, SARS-CoV-2; IF, immunofluorescence; N, the denominator used to inform the median, low and high values; PCR, polymerase chain reaction; SoC, standard of care; sp, species; TB, tuberculosis; UK, United Kingdom.

**Supplementary Table 4. Diagnostic tests used for immunocompetent patients admitted with CAP in the UK (Panel 1, Panel 3 and Panel 1+3 strategies)**

| **Diagnostic test** | **Panel 1**  **CAP patients tested, %** | | | | **Panel 3**  **CAP patients tested, %** | | | | **Panel 1+3**  **CAP patients tested, %** | | |
| --- | --- | --- | --- | --- | --- | --- | --- | --- | --- | --- | --- |
|  | **Median** | **Low value** | **High value** | **Median** | | **Low value** | **High value** | **Median** | | **Low value** | **High value** |
| Respiratory 1 Panel | 100 | 50 | 100 | 0 | | 0 | 0 | 100 | | 50 | 100 |
| Respiratory 3 Panel | 0 | 0 | 0 | 20 | | 10 | 100 | 20 | | 10 | 100 |
| Pleural fluid culture | 5 | 0 | 30 | 5 | | 0 | 30 | 5 | | 0 | 30 |
| Blood culture | 80 | 15 | 100 | 80 | | 15 | 100 | 80 | | 15 | 100 |
| Sputum culture | 50 | 10 | 100 | 50 | | 10 | 100 | 50 | | 10 | 100 |
| BAL culture | 5 | 0 | 20 | 5 | | 0 | 20 | 5 | | 0 | 20 |
| COVID-19 PCR | 0 | 0 | 0 | 100 | | 0 | 100 | 0 | | 0 | 0 |
| Respiratory virus PCR screen (respiratory sample) | 0 | 0 | 0 | 60 | | 0 | 100 | 0 | | 0 | 0 |
| Legionella culture (sputum sample) | 1 | 0 | 90 | 1 | | 0 | 90 | 1 | | 0 | 90 |
| Legionella PCR (sputum sample) | 0 | 0 | 40 | 0 | | 0 | 0 | 0 | | 0 | 0 |
| Legionella culture (BAL sample) | 1 | 0 | 10 | 1 | | 0 | 10 | 1 | | 0 | 10 |
| Legionella PCR (BAL sample) | 0 | 0 | 10 | 0 | | 0 | 0 | 0 | | 0 | 0 |
| Legionella PCR (pleural fluid sample) | 0 | 0 | 10 | 0 | | 0 | 0 | 0 | | 0 | 0 |
| Legionella urinary antigen test | 15 | 0 | 90 | 0 | | 0 | 0 | 0 | | 0 | 0 |
| *Streptococcus pneumoniae* urinary antigen test | 20 | 0 | 90 | 0 | | 0 | 0 | 0 | | 0 | 0 |
| Chlamydophila sp PCR (respiratory sample) | 1 | 0 | 15 | 1 | | 0 | 15 | 1 | | 0 | 15 |
| Chlamydophila sp serology | 0 | 0 | 10 | 0 | | 0 | 10 | 0 | | 0 | 10 |
| Mycobacterium culture (BAL sample) | 3 | 0 | 50 | 3 | | 0 | 50 | 3 | | 0 | 50 |
| Mycobacterium culture (sputum sample) | 5 | 0 | 50 | 5 | | 0 | 50 | 5 | | 0 | 50 |
| Mycobacterium PCR | 2 | 0 | 20 | 2 | | 0 | 20 | 2 | | 0 | 20 |
| *Mycoplasma pneumoniae* PCR (respiratory sample) | 0 | 0 | 0 | 0 | | 0 | 0 | 0 | | 0 | 0 |
| *Mycoplasma pneumoniae* serology | 0 | 0 | 0 | 0 | | 0 | 0 | 0 | | 0 | 0 |

Abbreviations: BAL, bronchoalveolar lavage, CAP, community acquired pneumonia; COVID-19, SARS-CoV-2; PCR, polymerase chain reaction; sp, species; UK, United Kingdom.

**Supplementary Table 5. Diagnostic tests used for immunocompromised patients admitted with CAP in the UK (Panel 1, Panel 3 and Panel 1+3 strategies)**

| **Diagnostic tests** | **Panel 1**  **CAP patients tested, %** | | | | **Panel 3**  **CAP patients tested, %** | | | | **Panel 1+3**  **CAP patients tested, %** | | |
| --- | --- | --- | --- | --- | --- | --- | --- | --- | --- | --- | --- |
|  | **Median** | **Low value** | **High value** | **Median** | | **Low value** | **High value** | **Median** | | **Low value** | **High value** |
| Respiratory 1 Panel | 100 | 50 | 100 | 0 | | 0 | 0 | 100 | | 50 | 100 |
| Respiratory 3 Panel | 0 | 0 | 0 | 30 | | 15 | 100 | 30 | | 15 | 100 |
| Blood culture | 80 | 20 | 100 | 80 | | 20 | 100 | 80 | | 20 | 100 |
| Sputum culture | 40 | 10 | 95 | 40 | | 10 | 95 | 40 | | 10 | 95 |
| BAL culture | 10 | 0 | 30 | 10 | | 0 | 30 | 10 | | 0 | 30 |
| Pleural fluid culture | 5 | 0 | 20 | 5 | | 0 | 20 | 5 | | 0 | 20 |
| COVID-19 PCR | 0 | 0 | 0 | 100 | | 0 | 100 | 0 | | 0 | 0 |
| Respiratory virus PCR screen | 0 | 0 | 0 | 40 | | 0 | 100 | 0 | | 0 | 0 |
| Legionella culture (sputum sample) | 0 | 0 | 90 | 0 | | 0 | 90 | 0 | | 0 | 90 |
| Legionella PCR (sputum sample) | 0 | 0 | 50 | 0 | | 0 | 0 | 0 | | 0 | 0 |
| Legionella culture (BAL sample) | 1 | 0 | 50 | 1 | | 0 | 50 | 1 | | 0 | 50 |
| Legionella PCR (BAL sample) | 0 | 0 | 20 | 0 | | 0 | 0 | 0 | | 0 | 0 |
| Legionella PCR (pleural fluid sample) | 0 | 0 | 20 | 0 | | 0 | 0 | 0 | | 0 | 0 |
| Legionella urinary antigen test | 30 | 0 | 100 | 0 | | 0 | 0 | 0 | | 0 | 0 |
| *Streptococcus pneumoniae* urinary antigen test | 30 | 0 | 100 | 0 | | 0 | 0 | 0 | | 0 | 0 |
| Chlamydophila sp PCR (respiratory sample) | 1 | 0 | 20 | 1 | | 0 | 20 | 1 | | 0 | 20 |
| Chlamydophila sp serology | 1 | 0 | 30 | 1 | | 0 | 30 | 1 | | 0 | 30 |
| Mycobacterium culture (BAL sample) | 8 | 0 | 75 | 8 | | 0 | 75 | 8 | | 0 | 75 |
| Mycobacterium culture (sputum sample) | 10 | 0 | 60 | 10 | | 0 | 60 | 10 | | 0 | 60 |
| Mycobacterium PCR | 8 | 0 | 40 | 8 | | 0 | 40 | 8 | | 0 | 40 |
| *Mycoplasma pneumoniae* PCR (respiratory sample) | 0 | 0 | 0 | 0 | | 0 | 0 | 0 | | 0 | 0 |
| *Mycoplasma pneumoniae* serology | 0 | 0 | 0 | 0 | | 0 | 0 | 0 | | 0 | 0 |

Table 5 continued on the next page.

Table 5 continued from the previous page.

| **Diagnostic tests** | **Panel 1**  **CAP patients tested, %** | | | | **Panel 3**  **CAP patients tested, %** | | | | **Panel 1+3**  **CAP patients tested, %** | | |
| --- | --- | --- | --- | --- | --- | --- | --- | --- | --- | --- | --- |
|  | **Median** | **Low value** | **High value** | **Median** | | **Low value** | **High value** | **Median** | | **Low value** | **High value** |
| Mycology culture (BAL sample) | 5 | 0 | 20 | 5 | | 0 | 20 | 5 | | 0 | 20 |
| Mycology culture (sputum sample) | 2 | 0 | 50 | 2 | | 0 | 50 | 2 | | 0 | 50 |
| Mycology PCR (BAL sample) | 1 | 0 | 40 | 1 | | 0 | 40 | 1 | | 0 | 40 |
| Mycology PCR (sputum sample) | 0 | 0 | 39 | 0 | | 0 | 39 | 0 | | 0 | 39 |
| *Pneumocystis jirovecii* PCR (BAL sample) | 10 | 0 | 49 | 0 | | 0 | 0 | 0 | | 0 | 0 |
| *Pneumocystis jirovecii* PCR (sputum sample) | 1 | 0 | 40 | 0 | | 0 | 0 | 0 | | 0 | 0 |
| *Pneumocystis jirovecii* IF (BAL sample) | 5 | 0 | 40 | 0 | | 0 | 0 | 0 | | 0 | 0 |
| *Pneumocystis jirovecii* IF (sputum sample) | 1 | 0 | 30 | 0 | | 0 | 0 | 0 | | 0 | 0 |
| Adenovirus screen | 0 | 0 | 30 | 0 | | 0 | 0 | 0 | | 0 | 0 |
| Aspergillus serum antigen | 3 | 0 | 40 | 3 | | 0 | 40 | 3 | | 0 | 40 |
| Cryptococcus serum antigen | 1 | 0 | 30 | 1 | | 0 | 30 | 1 | | 0 | 30 |
| Cytomegalovirus PCR (BAL sample) | 0 | 0 | 30 | 0 | | 0 | 30 | 0 | | 0 | 30 |
| Cytomegalovirus PCR (sputum sample) | 0 | 0 | 30 | 0 | | 0 | 30 | 0 | | 0 | 30 |
| Cytomegalovirus PCR serum | 0 | 0 | 30 | 0 | | 0 | 30 | 0 | | 0 | 30 |
| Epstein-Barr Virus screen | 5 | 0 | 30 | 5 | | 0 | 30 | 5 | | 0 | 30 |
| Nocardia culture | 0 | 0 | 10 | 0 | | 0 | 10 | 0 | | 0 | 10 |
| Non-TB mycobacteria PCR (BAL sample) | 3 | 0 | 40 | 3 | | 0 | 40 | 3 | | 0 | 40 |

Abbreviations: BAL, bronchoalveolar lavage, CAP, community acquired pneumonia; COVID-19, SARS-CoV-2; IF, immunofluorescence; PCR, polymerase chain reaction; SoC, standard of care; sp, species; TB, tuberculosis; UK, United Kingdom.

**Supplementary Table 6. Diagnostic tests used for immunocompetent patients admitted with CAP in France (SoC)**

| **Diagnostic test** | **CAP patients tested, %** | | | | | **Cost (per unit) to health service, €** | | | |
| --- | --- | --- | --- | --- | --- | --- | --- | --- | --- |
|  | **Median** | **Low value** | **High value** | **N** | **Median** | | **Low value** | **High value** | **N** |
| Respiratory 1 Panel | - | - | - | - | 23.88 | | 21.49 | 28.65 | - |
| Respiratory 3 Panel | - | - | - | - | 23.88 | | 21.49 | 28.65 | - |
| Pleural fluid culture | 30 | 0 | 100 | 15 | 30.00 | | 10.00 | 75.00 | 11 |
| Blood culture | 100 | 10 | 100 | 15 | 25.00 | | 5.00 | 55.00 | 13 |
| Sputum culture | 65 | 10 | 100 | 15 | 30.00 | | 10.00 | 59.00 | 11 |
| BAL culture | 35 | 20 | 89 | 15 | 30.00 | | 10.00 | 85.00 | 11 |
| COVID-19 PCR | 100 | 10 | 100 | 15 | 25.00 | | 10.00 | 110.00 | 13 |
| Respiratory virus PCR screen (respiratory sample) | 60 | 0 | 100 | 13 | 30.00 | | 10.00 | 110.00 | 10 |
| Legionella culture (sputum sample) | 25 | 0 | 100 | 15 | 42.50 | | 10.00 | 80.00 | 8 |
| Legionella PCR (sputum sample) | 25 | 0 | 100 | 15 | 49.00 | | 10.00 | 110.00 | 9 |
| Legionella culture (BAL sample) | 30 | 0 | 100 | 15 | 42.50 | | 10.00 | 80.00 | 8 |
| Legionella PCR (BAL sample) | 40 | 0 | 100 | 15 | 49.00 | | 10.00 | 110.00 | 9 |
| Legionella PCR (pleural fluid sample) | 20 | 0 | 100 | 15 | 49.00 | | 10.00 | 110.00 | 9 |
| Legionella urinary antigen test | 100 | 5 | 100 | 15 | 20.00 | | 8.00 | 100.00 | 10 |
| *Streptococcus pneumoniae* urinary antigen test | 100 | 5 | 100 | 15 | 30.00 | | 10.00 | 60.00 | 11 |
| Chlamydophila sp PCR (respiratory sample) | 40 | 0 | 100 | 15 | 27.50 | | 5.00 | 110.00 | 10 |
| Chlamydophila sp serology | 40 | 0 | 100 | 15 | 24.00 | | 10.00 | 65.00 | 9 |
| Mycobacterium culture (BAL sample) | 25 | 0 | 100 | 15 | 32.50 | | 10.00 | 46.00 | 8 |
| Mycobacterium culture (sputum sample) | 25 | 0 | 100 | 15 | 32.50 | | 10.00 | 46.00 | 8 |
| Mycobacterium PCR | 20 | 0 | 100 | 15 | 30.00 | | 10.00 | 55.00 | 8 |
| *Mycoplasma pneumoniae* PCR (respiratory sample) | 25 | 0 | 100 | 15 | 30.00 | | 8.00 | 110.00 | 9 |
| *Mycoplasma pneumoniae* serology | 20 | 0 | 100 | 15 | 26.00 | | 10.00 | 90.00 | 8 |

Abbreviations: BAL, bronchoalveolar lavage, CAP, community acquired pneumonia; COVID-19, SARS-CoV-2; N, the denominator used to inform the median, low and high values; PCR, polymerase chain reaction; SoC, standard of care; sp, species; UK, United Kingdom.

**Supplementary Table 7. Diagnostic tests used for immunocompromised patients admitted with CAP in France (SoC)**

| **Diagnostic test** | **CAP patients tested, %** | | | | | **Cost (per unit) to health service, €** | | | |
| --- | --- | --- | --- | --- | --- | --- | --- | --- | --- |
|  | **Median** | **Low value** | **High value** | **N** | **Median** | | **Low value** | **High value** | **N** |
| Blood culture | 100 | 10 | 100 | 15 | 25.00 | | 5.00 | 55.00 | 13 |
| Sputum culture | 80 | 20 | 100 | 15 | 30.00 | | 10.00 | 59.00 | 11 |
| BAL culture | 45 | 20 | 100 | 15 | 30.00 | | 10.00 | 85.00 | 11 |
| Pleural fluid culture | 30 | 10 | 70 | 15 | 30.00 | | 10.00 | 75.00 | 11 |
| COVID-19 PCR | 100 | 20 | 100 | 15 | 25.00 | | 10.00 | 110.00 | 13 |
| Respiratory virus PCR screen | 50 | 0 | 100 | 10 | 30.00 | | 10.00 | 110.00 | 10 |
| Legionella culture (sputum sample) | 25 | 0 | 100 | 15 | 42.50 | | 10.00 | 80.00 | 8 |
| Legionella PCR (sputum sample) | 30 | 0 | 100 | 15 | 49.00 | | 10.00 | 110.00 | 9 |
| Legionella culture (BAL sample) | 20 | 0 | 70 | 15 | 42.50 | | 10.00 | 80.00 | 8 |
| Legionella PCR (BAL sample) | 20 | 0 | 100 | 15 | 49.00 | | 10.00 | 110.00 | 9 |
| Legionella PCR (pleural fluid sample) | 10 | 0 | 100 | 15 | 49.00 | | 10.00 | 110.00 | 9 |
| Legionella urinary antigen test | 80 | 0 | 100 | 15 | 20.00 | | 8.00 | 100.00 | 10 |
| *Streptococcus pneumoniae* urinary antigen test | 65 | 0 | 100 | 15 | 30.00 | | 10.00 | 60.00 | 11 |
| Chlamydophila sp PCR (respiratory sample) | 25 | 0 | 100 | 15 | 27.50 | | 5.00 | 110.00 | 10 |
| Chlamydophila sp serology | 65 | 0 | 100 | 15 | 24.00 | | 10.00 | 65.00 | 9 |
| Mycobacterium culture (BAL sample) | 50 | 0 | 100 | 15 | 32.50 | | 10.00 | 46.00 | 8 |
| Mycobacterium culture (sputum sample) | 30 | 0 | 100 | 15 | 32.50 | | 10.00 | 46.00 | 8 |
| Mycobacterium PCR | 24 | 0 | 100 | 15 | 30.00 | | 10.00 | 55.00 | 8 |
| *Mycoplasma pneumoniae* PCR (respiratory sample) | 20 | 0 | 100 | 15 | 30.00 | | 8.00 | 110.00 | 9 |
| *Mycoplasma pneumoniae* serology | 10 | 0 | 100 | 15 | 26.00 | | 10.00 | 90.00 | 8 |
| Mycology culture (BAL sample) | 47 | 0 | 100 | 15 | 30.00 | | 10.00 | 60.00 | 9 |
| Mycology culture (sputum sample) | 21 | 0 | 100 | 15 | 30.00 | | 10.00 | 60.00 | 9 |
| Mycology PCR (BAL sample) | 29 | 0 | 100 | 15 | 29.50 | | 10.00 | 50.00 | 8 |
| Mycology PCR (sputum sample) | 10 | 0 | 100 | 15 | 29.50 | | 10.00 | 50.00 | 8 |

Table 7 continued on the next page.

Table 7 continued from the previous page.

| **Diagnostic test** | **CAP patients tested, %** | | | | | **Cost (per unit) to health service, €** | | | |
| --- | --- | --- | --- | --- | --- | --- | --- | --- | --- |
|  | **Median** | **Low value** | **High value** | **N** | **Median** | | **Low value** | **High value** | **N** |
| *Pneumocystis jirovecii* PCR (BAL sample) | 50 | 0 | 100 | 15 | 27.50 | | 10.00 | 80.00 | 8 |
| *Pneumocystis jirovecii* PCR (sputum sample) | 20 | 0 | 100 | 15 | 27.50 | | 10.00 | 80.00 | 8 |
| *Pneumocystis jirovecii* IF (BAL sample) | 25 | 0 | 100 | 15 | 30.00 | | 10.00 | 120.00 | 8 |
| *Pneumocystis jirovecii* IF (sputum sample) | 20 | 0 | 100 | 15 | 30.00 | | 10.00 | 120.00 | 8 |
| Adenovirus screen | 20 | 0 | 100 | 15 | 27.50 | | 10.00 | 110.00 | 12 |
| Aspergillus serum antigen | 50 | 5 | 100 | 15 | 30.00 | | 15.00 | 65.00 | 11 |
| Cryptococcus serum antigen | 20 | 0 | 100 | 15 | 35.00 | | 5.00 | 65.00 | 9 |
| Cytomegalovirus PCR (BAL sample) | 20 | 0 | 100 | 15 | 30.00 | | 10.00 | 80.00 | 9 |
| Cytomegalovirus PCR (sputum sample) | 10 | 0 | 100 | 15 | 30.00 | | 10.00 | 80.00 | 9 |
| Cytomegalovirus PCR serum | 20 | 0 | 100 | 15 | 30.00 | | 10.00 | 80.00 | 9 |
| Epstein-Barr Virus screen | 20 | 0 | 100 | 15 | 20.00 | | 10.00 | 60.00 | 9 |
| Nocardia culture | 20 | 0 | 100 | 15 | 20.00 | | 5.00 | 85.00 | 9 |
| Non-TB mycobacteria PCR (BAL sample) | 20 | 0 | 100 | 15 | 25.00 | | 10.00 | 67.00 | 8 |

Abbreviations: BAL, bronchoalveolar lavage, CAP, community acquired pneumonia; COVID-19, SARS-CoV-2; IF, immunofluorescence; N, the denominator used to inform the median, low and high values; PCR, polymerase chain reaction; SoC, standard of care; sp, species; TB, tuberculosis; UK, United Kingdom.

**Supplementary Table 8. Diagnostic tests used for immunocompetent patients admitted with CAP in France (Panel 1, Panel 3 and Panel 1+3 strategies)**

| **Diagnostic test** | **Panel 1**  **CAP patients tested, %** | | | | **Panel 3**  **CAP patients tested, %** | | | | **Panel 1+3**  **CAP patients tested, %** | | |
| --- | --- | --- | --- | --- | --- | --- | --- | --- | --- | --- | --- |
|  | **Median** | **Low value** | **High value** | **Median** | | **Low value** | **High value** | **Median** | | **Low value** | **High value** |
| Respiratory 1 Panel | 100 | 50 | 100 | 0 | | 0 | 0 | 100 | | 50 | 100 |
| Respiratory 3 Panel | 0 | 0 | 0 | 100 | | 50 | 100 | 100 | | 50 | 100 |
| Pleural fluid culture | 30 | 0 | 100 | 30 | | 0 | 100 | 30 | | 0 | 100 |
| Blood culture | 100 | 10 | 100 | 100 | | 10 | 100 | 100 | | 10 | 100 |
| Sputum culture | 65 | 10 | 100 | 65 | | 10 | 100 | 65 | | 10 | 100 |
| BAL culture | 35 | 20 | 89 | 35 | | 20 | 89 | 35 | | 20 | 89 |
| COVID-19 PCR | 0 | 0 | 0 | 100 | | 10 | 100 | 0 | | 0 | 0 |
| Respiratory virus PCR screen (respiratory sample) | 0 | 0 | 0 | 60 | | 0 | 100 | 0 | | 0 | 0 |
| Legionella culture (sputum sample) | 25 | 0 | 100 | 25 | | 0 | 100 | 25 | | 0 | 100 |
| Legionella PCR (sputum sample) | 25 | 0 | 100 | 0 | | 0 | 0 | 0 | | 0 | 0 |
| Legionella culture (BAL sample) | 30 | 0 | 100 | 30 | | 0 | 100 | 30 | | 0 | 100 |
| Legionella PCR (BAL sample) | 40 | 0 | 100 | 0 | | 0 | 0 | 0 | | 0 | 0 |
| Legionella PCR (pleural fluid sample) | 20 | 0 | 100 | 0 | | 0 | 0 | 0 | | 0 | 0 |
| Legionella urinary antigen test | 100 | 5 | 100 | 0 | | 0 | 0 | 0 | | 0 | 0 |
| *Streptococcus pneumoniae* urinary antigen test | 100 | 5 | 100 | 0 | | 0 | 0 | 0 | | 0 | 0 |
| Chlamydophila sp PCR (respiratory sample) | 40 | 0 | 100 | 40 | | 0 | 100 | 40 | | 0 | 100 |
| Chlamydophila sp serology | 40 | 0 | 100 | 40 | | 0 | 100 | 40 | | 0 | 100 |
| Mycobacterium culture (BAL sample) | 25 | 0 | 100 | 25 | | 0 | 100 | 25 | | 0 | 100 |
| Mycobacterium culture (sputum sample) | 25 | 0 | 100 | 25 | | 0 | 100 | 25 | | 0 | 100 |
| Mycobacterium PCR | 20 | 0 | 100 | 20 | | 0 | 100 | 20 | | 0 | 100 |
| *Mycoplasma pneumoniae* PCR (respiratory sample) | 0 | 0 | 0 | 0 | | 0 | 0 | 0 | | 0 | 0 |
| *Mycoplasma pneumoniae* serology | 0 | 0 | 0 | 0 | | 0 | 0 | 0 | | 0 | 0 |

Abbreviations: BAL, bronchoalveolar lavage, CAP, community acquired pneumonia; COVID-19, SARS-CoV-2; PCR, polymerase chain reaction; sp, species; UK, United Kingdom.

**Supplementary Table 9. Diagnostic tests used for immunocompromised patients admitted with CAP in France (Panel 1, Panel 3 and Panel 1+3 strategies)**

| **Diagnostic tests** | **Panel 1**  **CAP patients tested, %** | | | | **Panel 3**  **CAP patients tested, %** | | | | **Panel 1+3**  **CAP patients tested, %** | | |
| --- | --- | --- | --- | --- | --- | --- | --- | --- | --- | --- | --- |
|  | **Median** | **Low value** | **High value** | **Median** | | **Low value** | **High value** | **Median** | | **Low value** | **High value** |
| Respiratory 1 Panel | 100 | 50 | 100 | 0 | | 0 | 0 | 100 | | 50 | 100 |
| Respiratory 3 Panel | 0 | 0 | 0 | 80 | | 40 | 100 | 80 | | 40 | 100 |
| Blood culture | 100 | 10 | 100 | 100 | | 10 | 100 | 100 | | 10 | 100 |
| Sputum culture | 80 | 20 | 100 | 80 | | 20 | 100 | 80 | | 20 | 100 |
| BAL culture | 45 | 20 | 100 | 45 | | 20 | 100 | 45 | | 20 | 100 |
| Pleural fluid culture | 30 | 10 | 70 | 30 | | 10 | 70 | 30 | | 10 | 70 |
| COVID-19 PCR | 0 | 0 | 0 | 100 | | 20 | 100 | 0 | | 0 | 0 |
| Respiratory virus PCR screen | 0 | 0 | 0 | 50 | | 0 | 100 | 0 | | 0 | 0 |
| Legionella culture (sputum sample) | 25 | 0 | 100 | 25 | | 0 | 100 | 25 | | 0 | 100 |
| Legionella PCR (sputum sample) | 30 | 0 | 100 | 0 | | 0 | 0 | 0 | | 0 | 0 |
| Legionella culture (BAL sample) | 20 | 0 | 70 | 20 | | 0 | 70 | 20 | | 0 | 70 |
| Legionella PCR (BAL sample) | 20 | 0 | 100 | 0 | | 0 | 0 | 0 | | 0 | 0 |
| Legionella PCR (pleural fluid sample) | 10 | 0 | 100 | 0 | | 0 | 0 | 0 | | 0 | 0 |
| Legionella urinary antigen test | 80 | 0 | 100 | 0 | | 0 | 0 | 0 | | 0 | 0 |
| *Streptococcus pneumoniae* urinary antigen test | 65 | 0 | 100 | 0 | | 0 | 0 | 0 | | 0 | 0 |
| Chlamydophila sp PCR (respiratory sample) | 25 | 0 | 100 | 25 | | 0 | 100 | 25 | | 0 | 100 |
| Chlamydophila sp serology | 65 | 0 | 100 | 65 | | 0 | 100 | 65 | | 0 | 100 |
| Mycobacterium culture (BAL sample) | 50 | 0 | 100 | 50 | | 0 | 100 | 50 | | 0 | 100 |
| Mycobacterium culture (sputum sample) | 30 | 0 | 100 | 30 | | 0 | 100 | 30 | | 0 | 100 |
| Mycobacterium PCR | 24 | 0 | 100 | 24 | | 0 | 100 | 24 | | 0 | 100 |
| *Mycoplasma pneumoniae* PCR (respiratory sample) | 0 | 0 | 0 | 0 | | 0 | 0 | 0 | | 0 | 0 |
| *Mycoplasma pneumoniae* serology | 0 | 0 | 0 | 0 | | 0 | 0 | 0 | | 0 | 0 |

Table 9 continued on the next page.

Table 9 continued from the previous page.

| **Diagnostic tests** | **Panel 1**  **CAP patients tested, %** | | | | **Panel 3**  **CAP patients tested, %** | | | | **Panel 1+3**  **CAP patients tested, %** | | |
| --- | --- | --- | --- | --- | --- | --- | --- | --- | --- | --- | --- |
|  | **Median** | **Low value** | **High value** | **Median** | | **Low value** | **High value** | **Median** | | **Low value** | **High value** |
| Mycology culture (BAL sample) | 47 | 0 | 100 | 47 | | 0 | 100 | 47 | | 0 | 100 |
| Mycology culture (sputum sample) | 21 | 0 | 100 | 21 | | 0 | 100 | 21 | | 0 | 100 |
| Mycology PCR (BAL sample) | 29 | 0 | 100 | 29 | | 0 | 100 | 29 | | 0 | 100 |
| Mycology PCR (sputum sample) | 10 | 0 | 100 | 10 | | 0 | 100 | 10 | | 0 | 100 |
| *Pneumocystis jirovecii* PCR (BAL sample) | 50 | 0 | 100 | 0 | | 0 | 0 | 0 | | 0 | 0 |
| *Pneumocystis jirovecii* PCR (sputum sample) | 20 | 0 | 100 | 0 | | 0 | 0 | 0 | | 0 | 0 |
| *Pneumocystis jirovecii* IF (BAL sample) | 25 | 0 | 100 | 0 | | 0 | 0 | 0 | | 0 | 0 |
| *Pneumocystis jirovecii* IF (sputum sample) | 20 | 0 | 100 | 0 | | 0 | 0 | 0 | | 0 | 0 |
| Adenovirus screen | 20 | 0 | 100 | 0 | | 0 | 0 | 0 | | 0 | 0 |
| Aspergillus serum antigen | 50 | 5 | 100 | 50 | | 5 | 100 | 50 | | 5 | 100 |
| Cryptococcus serum antigen | 20 | 0 | 100 | 20 | | 0 | 100 | 20 | | 0 | 100 |
| Cytomegalovirus PCR (BAL sample) | 20 | 0 | 100 | 20 | | 0 | 100 | 20 | | 0 | 100 |
| Cytomegalovirus PCR (sputum sample) | 10 | 0 | 100 | 10 | | 0 | 100 | 10 | | 0 | 100 |
| Cytomegalovirus PCR serum | 20 | 0 | 100 | 20 | | 0 | 100 | 20 | | 0 | 100 |
| Epstein-Barr Virus screen | 20 | 0 | 100 | 20 | | 0 | 100 | 20 | | 0 | 100 |
| Nocardia culture | 20 | 0 | 100 | 20 | | 0 | 100 | 20 | | 0 | 100 |
| Non-TB mycobacteria PCR (BAL sample) | 20 | 0 | 100 | 20 | | 0 | 100 | 20 | | 0 | 100 |

Abbreviations: BAL, bronchoalveolar lavage, CAP, community acquired pneumonia; COVID-19, SARS-CoV-2; IF, immunofluorescence; PCR, polymerase chain reaction; SoC, standard of care; sp, species; TB, tuberculosis; UK, United Kingdom.

**Supplementary Table 10. Diagnostic tests used for immunocompetent patients admitted with CAP in Spain (SoC)**

| **Diagnostic test** | **CAP patients tested, %** | | | | | **Cost (per unit) to health service, €** | | | |
| --- | --- | --- | --- | --- | --- | --- | --- | --- | --- |
|  | **Median** | **Low value** | **High value** | **N** | **Median** | | **Low value** | **High value** | **N** |
| Respiratory 1 Panel | - | - | - | - | 23.88 | | 21.49 | 28.65 | - |
| Respiratory 3 Panel | - | - | - | - | 23.88 | | 21.49 | 28.65 | - |
| Pleural fluid culture | 10 | 3 | 34 | 15 | 25.00 | | 5.00 | 100.00 | 14 |
| Blood culture | 70 | 19 | 90 | 15 | 20.00 | | 4.00 | 100.00 | 15 |
| Sputum culture | 50 | 15 | 90 | 15 | 20.00 | | 2.00 | 100.00 | 15 |
| BAL culture | 15 | 5 | 60 | 15 | 20.00 | | 5.00 | 100.00 | 15 |
| COVID-19 PCR | 100 | 12 | 100 | 15 | 30.00 | | 4.00 | 138.33 | 14 |
| Respiratory virus PCR screen (respiratory sample) | 45 | 0 | 100 | 12 | 30.00 | | 10.00 | 150.00 | 14 |
| Legionella culture (sputum sample) | 10 | 0 | 60 | 15 | 32.50 | | 5.00 | 100.00 | 12 |
| Legionella PCR (sputum sample) | 5 | 0 | 75 | 15 | 35.00 | | 12.00 | 138.33 | 12 |
| Legionella culture (BAL sample) | 10 | 0 | 21 | 15 | 32.50 | | 5.00 | 100.00 | 12 |
| Legionella PCR (BAL sample) | 2 | 0 | 20 | 15 | 35.00 | | 12.00 | 138.33 | 12 |
| Legionella PCR (pleural fluid sample) | 1 | 0 | 20 | 15 | 35.00 | | 12.00 | 138.33 | 12 |
| Legionella urinary antigen test | 80 | 10 | 100 | 15 | 10.00 | | 2.00 | 70.00 | 13 |
| *Streptococcus pneumoniae* urinary antigen test | 70 | 10 | 100 | 15 | 15.00 | | 2.00 | 100.00 | 13 |
| Chlamydophila sp PCR (respiratory sample) | 5 | 0 | 50 | 15 | 34.00 | | 10.00 | 138.33 | 11 |
| Chlamydophila sp serology | 10 | 0 | 50 | 15 | 20.00 | | 4.00 | 168.38 | 13 |
| Mycobacterium culture (BAL sample) | 10 | 0 | 58 | 15 | 45.00 | | 5.00 | 100.00 | 13 |
| Mycobacterium culture (sputum sample) | 20 | 5 | 70 | 15 | 45.00 | | 5.00 | 100.00 | 13 |
| Mycobacterium PCR | 10 | 0 | 30 | 15 | 46.00 | | 15.00 | 179.10 | 13 |
| *Mycoplasma pneumoniae* PCR (respiratory sample) | 5 | 0 | 50 | 15 | 49.00 | | 15.00 | 138.33 | 11 |
| *Mycoplasma pneumoniae* serology | 14 | 0 | 50 | 15 | 20.00 | | 5.00 | 168.38 | 13 |

Abbreviations: BAL, bronchoalveolar lavage, CAP, community acquired pneumonia; COVID-19, SARS-CoV-2; N, the denominator used to inform the median, low and high values; PCR, polymerase chain reaction; SoC, standard of care; sp, species; UK, United Kingdom.

**Supplementary Table 11. Diagnostic tests used for immunocompromised patients admitted with CAP in Spain (SoC)**

| **Diagnostic test** | **CAP patients tested, %** | | | | | **Cost (per unit) to health service, €** | | | |
| --- | --- | --- | --- | --- | --- | --- | --- | --- | --- |
|  | **Median** | **Low value** | **High value** | **N** | **Median** | | **Low value** | **High value** | **N** |
| Blood culture | 90 | 40 | 100 | 15 | 20.00 | | 4.00 | 100.00 | 15 |
| Sputum culture | 80 | 40 | 90 | 15 | 20.00 | | 2.00 | 100.00 | 13 |
| BAL culture | 40 | 10 | 60 | 15 | 20.00 | | 5.00 | 100.00 | 13 |
| Pleural fluid culture | 15 | 5 | 50 | 15 | 25.00 | | 5.00 | 100.00 | 14 |
| COVID-19 PCR | 100 | 78 | 100 | 15 | 30.00 | | 4.00 | 138.33 | 14 |
| Respiratory virus PCR screen | 70 | 0 | 100 | 11 | 30.00 | | 10.00 | 150.00 | 14 |
| Legionella culture (sputum sample) | 15 | 0 | 80 | 15 | 32.50 | | 5.00 | 100.00 | 12 |
| Legionella PCR (sputum sample) | 5 | 0 | 70 | 15 | 35.00 | | 12.00 | 138.33 | 12 |
| Legionella culture (BAL sample) | 10 | 0 | 57 | 15 | 32.50 | | 5.00 | 100.00 | 12 |
| Legionella PCR (BAL sample) | 7 | 0 | 65 | 15 | 35.00 | | 12.00 | 138.33 | 12 |
| Legionella PCR (pleural fluid sample) | 5 | 0 | 45 | 15 | 35.00 | | 12.00 | 138.33 | 12 |
| Legionella urinary antigen test | 89 | 25 | 100 | 15 | 10.00 | | 2.00 | 70.00 | 13 |
| *Streptococcus pneumoniae* urinary antigen test | 95 | 40 | 100 | 15 | 15.00 | | 2.00 | 100.00 | 13 |
| Chlamydophila sp PCR (respiratory sample) | 10 | 0 | 50 | 15 | 34.00 | | 10.00 | 138.33 | 11 |
| Chlamydophila sp serology | 20 | 0 | 50 | 15 | 20.00 | | 4.00 | 168.38 | 13 |
| Mycobacterium culture (BAL sample) | 19 | 0 | 50 | 15 | 45.00 | | 5.00 | 100.00 | 13 |
| Mycobacterium culture (sputum sample) | 25 | 5 | 70 | 15 | 45.00 | | 5.00 | 100.00 | 13 |
| Mycobacterium PCR | 15 | 0 | 50 | 15 | 46.00 | | 15.00 | 179.10 | 13 |
| *Mycoplasma pneumoniae* PCR (respiratory sample) | 7 | 0 | 50 | 15 | 49.00 | | 15.00 | 138.33 | 11 |
| *Mycoplasma pneumoniae* serology | 10 | 5 | 90 | 15 | 20.00 | | 5.00 | 168.38 | 13 |
| Mycology culture (BAL sample) | 12 | 0 | 67 | 15 | 20.00 | | 5.00 | 100.00 | 13 |
| Mycology culture (sputum sample) | 10 | 3 | 70 | 15 | 20.00 | | 5.00 | 100.00 | 13 |
| Mycology PCR (BAL sample) | 10 | 0 | 50 | 15 | 30.00 | | 20.00 | 80.00 | 9 |
| Mycology PCR (sputum sample) | 5 | 0 | 80 | 15 | 30.00 | | 20.00 | 80.00 | 9 |

Table 11 continued on the next page.

Table 11 continued from the previous page.

| **Diagnostic test** | **CAP patients tested, %** | | | | | **Cost (per unit) to health service, €** | | | |
| --- | --- | --- | --- | --- | --- | --- | --- | --- | --- |
|  | **Median** | **Low value** | **High value** | **N** | **Median** | | **Low value** | **High value** | **N** |
| *Pneumocystis jirovecii* PCR (BAL sample) | 10 | 0 | 80 | 15 | 31.00 | | 15.00 | 100.00 | 12 |
| *Pneumocystis jirovecii* PCR (sputum sample) | 10 | 0 | 80 | 15 | 31.00 | | 15.00 | 100.00 | 12 |
| *Pneumocystis jirovecii* IF (BAL sample) | 10 | 0 | 80 | 15 | 30.00 | | 10.00 | 168.38 | 12 |
| *Pneumocystis jirovecii* IF (sputum sample) | 9 | 0 | 80 | 15 | 30.00 | | 10.00 | 168.38 | 12 |
| Adenovirus screen | 5 | 0 | 50 | 15 | 20.00 | | 3.00 | 50.00 | 11 |
| Aspergillus serum antigen | 15 | 2 | 70 | 15 | 25.00 | | 10.00 | 50.00 | 12 |
| Cryptococcus serum antigen | 5 | 0 | 50 | 15 | 20.00 | | 5.00 | 168.38 | 12 |
| Cytomegalovirus PCR (BAL sample) | 10 | 3 | 50 | 15 | 20.00 | | 10.00 | 179.10 | 13 |
| Cytomegalovirus PCR (sputum sample) | 5 | 0 | 60 | 15 | 20.00 | | 10.00 | 179.10 | 13 |
| Cytomegalovirus PCR serum | 4 | 0 | 67 | 15 | 20.00 | | 10.00 | 179.10 | 13 |
| Epstein-Barr Virus screen | 10 | 2 | 60 | 15 | 19.00 | | 3.97 | 40.00 | 12 |
| Nocardia culture | 10 | 0 | 70 | 15 | 20.00 | | 5.00 | 100.00 | 13 |
| Non-TB mycobacteria PCR (BAL sample) | 10 | 0 | 70 | 15 | 33.50 | | 10.00 | 157.26 | 12 |

Abbreviations: BAL, bronchoalveolar lavage, CAP, community acquired pneumonia; COVID-19, SARS-CoV-2; IF, immunofluorescence; N, the denominator used to inform the median, low and high values; PCR, polymerase chain reaction; SoC, standard of care; sp, species; TB, tuberculosis; UK, United Kingdom.

**Supplementary Table 12. Diagnostic tests used for immunocompetent patients admitted with CAP in Spain (Panel 1, Panel 3 and Panel 1+3 strategies)**

| **Diagnostic test** | **Panel 1**  **CAP patients tested, %** | | | | **Panel 3**  **CAP patients tested, %** | | | | **Panel 1+3**  **CAP patients tested, %** | | |
| --- | --- | --- | --- | --- | --- | --- | --- | --- | --- | --- | --- |
|  | **Median** | **Low value** | **High value** | **Median** | | **Low value** | **High value** | **Median** | | **Low value** | **High value** |
| Respiratory 1 Panel | 100 | 50 | 100 | 0 | | 0 | 0 | 100 | | 50 | 100 |
| Respiratory 3 Panel | 0 | 0 | 0 | 80 | | 40 | 100 | 80 | | 40 | 100 |
| Pleural fluid culture | 10 | 3 | 34 | 10 | | 3 | 34 | 10 | | 3 | 34 |
| Blood culture | 70 | 19 | 90 | 70 | | 19 | 90 | 70 | | 19 | 90 |
| Sputum culture | 50 | 15 | 90 | 50 | | 15 | 90 | 50 | | 15 | 90 |
| BAL culture | 15 | 5 | 60 | 15 | | 5 | 60 | 15 | | 5 | 60 |
| COVID-19 PCR | 0 | 0 | 0 | 100 | | 12 | 100 | 0 | | 0 | 0 |
| Respiratory virus PCR screen (respiratory sample) | 0 | 0 | 0 | 45 | | 0 | 100 | 0 | | 0 | 0 |
| Legionella culture (sputum sample) | 10 | 0 | 60 | 10 | | 0 | 60 | 10 | | 0 | 60 |
| Legionella PCR (sputum sample) | 5 | 0 | 75 | 0 | | 0 | 0 | 0 | | 0 | 0 |
| Legionella culture (BAL sample) | 10 | 0 | 21 | 10 | | 0 | 21 | 10 | | 0 | 21 |
| Legionella PCR (BAL sample) | 2 | 0 | 20 | 0 | | 0 | 0 | 0 | | 0 | 0 |
| Legionella PCR (pleural fluid sample) | 1 | 0 | 20 | 0 | | 0 | 0 | 0 | | 0 | 0 |
| Legionella urinary antigen test | 80 | 10 | 100 | 0 | | 0 | 0 | 0 | | 0 | 0 |
| *Streptococcus pneumoniae* urinary antigen test | 70 | 10 | 100 | 0 | | 0 | 0 | 0 | | 0 | 0 |
| Chlamydophila sp PCR (respiratory sample) | 5 | 0 | 50 | 5 | | 0 | 50 | 5 | | 0 | 50 |
| Chlamydophila sp serology | 10 | 0 | 50 | 10 | | 0 | 50 | 10 | | 0 | 50 |
| Mycobacterium culture (BAL sample) | 10 | 0 | 58 | 10 | | 0 | 58 | 10 | | 0 | 58 |
| Mycobacterium culture (sputum sample) | 20 | 5 | 70 | 20 | | 5 | 70 | 20 | | 5 | 70 |
| Mycobacterium PCR | 10 | 0 | 30 | 10 | | 0 | 30 | 10 | | 0 | 30 |
| *Mycoplasma pneumoniae* PCR (respiratory sample) | 0 | 0 | 0 | 0 | | 0 | 0 | 0 | | 0 | 0 |
| *Mycoplasma pneumoniae* serology | 0 | 0 | 0 | 0 | | 0 | 0 | 0 | | 0 | 0 |

Abbreviations: BAL, bronchoalveolar lavage, CAP, community acquired pneumonia; COVID-19, SARS-CoV-2; PCR, polymerase chain reaction; sp, species; UK, United Kingdom.

**Supplementary Table 13. Diagnostic tests used for immunocompromised patients admitted with CAP in Spain (Panel 1, Panel 3 and Panel 1+3 strategies)**

| **Diagnostic tests** | **Panel 1**  **CAP patients tested, %** | | | | **Panel 3**  **CAP patients tested, %** | | | | **Panel 1+3**  **CAP patients tested, %** | | |
| --- | --- | --- | --- | --- | --- | --- | --- | --- | --- | --- | --- |
|  | **Median** | **Low value** | **High value** | **Median** | | **Low value** | **High value** | **Median** | | **Low value** | **High value** |
| Respiratory 1 Panel | 100 | 50 | 100 | 0 | | 0 | 0 | 100 | | 50 | 100 |
| Respiratory 3 Panel | 0 | 0 | 0 | 95 | | 48 | 100 | 95 | | 48 | 100 |
| Blood culture | 90 | 40 | 100 | 90 | | 40 | 100 | 90 | | 40 | 100 |
| Sputum culture | 80 | 40 | 90 | 80 | | 40 | 90 | 80 | | 40 | 90 |
| BAL culture | 40 | 10 | 60 | 40 | | 10 | 60 | 40 | | 10 | 60 |
| Pleural fluid culture | 15 | 5 | 50 | 15 | | 5 | 50 | 15 | | 5 | 50 |
| COVID-19 PCR | 0 | 0 | 0 | 100 | | 78 | 100 | 0 | | 0 | 0 |
| Respiratory virus PCR screen | 0 | 0 | 0 | 70 | | 0 | 100 | 0 | | 0 | 0 |
| Legionella culture (sputum sample) | 15 | 0 | 80 | 15 | | 0 | 80 | 15 | | 0 | 80 |
| Legionella PCR (sputum sample) | 5 | 0 | 70 | 0 | | 0 | 0 | 0 | | 0 | 0 |
| Legionella culture (BAL sample) | 10 | 0 | 57 | 10 | | 0 | 57 | 10 | | 0 | 57 |
| Legionella PCR (BAL sample) | 7 | 0 | 65 | 0 | | 0 | 0 | 0 | | 0 | 0 |
| Legionella PCR (pleural fluid sample) | 5 | 0 | 45 | 0 | | 0 | 0 | 0 | | 0 | 0 |
| Legionella urinary antigen test | 89 | 25 | 100 | 0 | | 0 | 0 | 0 | | 0 | 0 |
| *Streptococcus pneumoniae* urinary antigen test | 95 | 40 | 100 | 0 | | 0 | 0 | 0 | | 0 | 0 |
| Chlamydophila sp PCR (respiratory sample) | 10 | 0 | 50 | 10 | | 0 | 50 | 10 | | 0 | 50 |
| Chlamydophila sp serology | 20 | 0 | 50 | 20 | | 0 | 50 | 20 | | 0 | 50 |
| Mycobacterium culture (BAL sample) | 19 | 0 | 50 | 19 | | 0 | 50 | 19 | | 0 | 50 |
| Mycobacterium culture (sputum sample) | 25 | 5 | 70 | 25 | | 5 | 70 | 25 | | 5 | 70 |
| Mycobacterium PCR | 15 | 0 | 50 | 15 | | 0 | 50 | 15 | | 0 | 50 |
| *Mycoplasma pneumoniae* PCR (respiratory sample) | 0 | 0 | 0 | 0 | | 0 | 0 | 0 | | 0 | 0 |
| *Mycoplasma pneumoniae* serology | 0 | 0 | 0 | 0 | | 0 | 0 | 0 | | 0 | 0 |

Table 13 continued on the next page.

Table 13 continued from the previous page.

| **Diagnostic tests** | **Panel 1**  **CAP patients tested, %** | | | | **Panel 3**  **CAP patients tested, %** | | | | **Panel 1+3**  **CAP patients tested, %** | | |
| --- | --- | --- | --- | --- | --- | --- | --- | --- | --- | --- | --- |
|  | **Median** | **Low value** | **High value** | **Median** | | **Low value** | **High value** | **Median** | | **Low value** | **High value** |
| Mycology culture (BAL sample) | 12 | 0 | 67 | 12 | | 0 | 67 | 12 | | 0 | 67 |
| Mycology culture (sputum sample) | 10 | 3 | 70 | 10 | | 3 | 70 | 10 | | 3 | 70 |
| Mycology PCR (BAL sample) | 10 | 0 | 50 | 10 | | 0 | 50 | 10 | | 0 | 50 |
| Mycology PCR (sputum sample) | 5 | 0 | 80 | 5 | | 0 | 80 | 5 | | 0 | 80 |
| *Pneumocystis jirovecii* PCR (BAL sample) | 10 | 0 | 80 | 0 | | 0 | 0 | 0 | | 0 | 0 |
| *Pneumocystis jirovecii* PCR (sputum sample) | 10 | 0 | 80 | 0 | | 0 | 0 | 0 | | 0 | 0 |
| *Pneumocystis jirovecii* IF (BAL sample) | 10 | 0 | 80 | 0 | | 0 | 0 | 0 | | 0 | 0 |
| *Pneumocystis jirovecii* IF (sputum sample) | 9 | 0 | 80 | 0 | | 0 | 0 | 0 | | 0 | 0 |
| Adenovirus screen | 5 | 0 | 50 | 0 | | 0 | 0 | 0 | | 0 | 0 |
| Aspergillus serum antigen | 15 | 2 | 70 | 15 | | 2 | 70 | 15 | | 2 | 70 |
| Cryptococcus serum antigen | 5 | 0 | 50 | 5 | | 0 | 50 | 5 | | 0 | 50 |
| Cytomegalovirus PCR (BAL sample) | 10 | 3 | 50 | 10 | | 3 | 50 | 10 | | 3 | 50 |
| Cytomegalovirus PCR (sputum sample) | 5 | 0 | 60 | 5 | | 0 | 60 | 5 | | 0 | 60 |
| Cytomegalovirus PCR serum | 4 | 0 | 67 | 4 | | 0 | 67 | 4 | | 0 | 67 |
| Epstein-Barr Virus screen | 10 | 2 | 60 | 10 | | 2 | 60 | 10 | | 2 | 60 |
| Nocardia culture | 10 | 0 | 70 | 10 | | 0 | 70 | 10 | | 0 | 70 |
| Non-TB mycobacteria PCR (BAL sample) | 10 | 0 | 70 | 10 | | 0 | 70 | 10 | | 0 | 70 |

Abbreviations: BAL, bronchoalveolar lavage, CAP, community acquired pneumonia; COVID-19, SARS-CoV-2; IF, immunofluorescence; PCR, polymerase chain reaction; SoC, standard of care; sp, species; TB, tuberculosis; UK, United Kingdom.

**Supplementary Table 14. Average cost of diagnostic testing per immunocompetent and immunocompromised patient admitted with CAP in the UK (£)**

| **Strategy** | **Median** | **Low** | **High** |
| --- | --- | --- | --- |
| **Immunocompetent patients** |  |  |  |
| SoC | £ 72.03 | £ 1.25 | £ 582.95 |
| Panel 1^1^ | £ 56.03 | £ 10.25 | £ 408.99 |
| Panel 3^1^ | £ 68.53 | £ 3.05 | £ 391.84 |
| Panel 1+3^1^ | £ 53.53 | £ 12.05 | £ 275.84 |
| Difference between SoC and Panel 1+3^2^ | -£ 18.50 | £ 10.80 | -£ 307.11 |
| **Immunocompromised patients** |  |  |  |
| SoC | £ 85.73 | £ 1.50 | £ 1,029.38 |
| Panel 1^1^ | £ 73.23 | £ 10.50 | £ 823.17 |
| Panel 3^1^ | £ 73.85 | £ 4.20 | £ 670.24 |
| Panel 1+3^1^ | £ 63.85 | £ 13.20 | £ 533.24 |
| Difference between SoC and Panel 1+3^2^ | -£ 21.88 | £ 11.70 | -£ 496.14 |

Abbreviations: SoC, standard of care. Equivalent data presented in Euros are presented in the main paper (Table 4).

^1^Panel 1 strategy refers to cost of using Panel 1 to replace some SoC tests plus the cost of diagnostic tests not replaced by Panel 1. The equivalent is true for Panel 3 strategy and for the Panel 1+3 strategy. ^2^A negative number here indicates that the Panel 1+3 strategy results in cost savings compared to SoC.

**Supplementary Table 15. Estimated monthly costs per hospital of diagnostic testing of patients admitted with CAP**

| **Variable** | **UK (€)** | **UK (£)** | **France** | **Spain** |
| --- | --- | --- | --- | --- |
| Immunocompetent patients (%) | 76.3% | 76.3% | 55.0% | 67.0% |
| Immunocompromised patients (%) | 23.8% | 23.8% | 45.0% | 33.0% |
| Total number of patients per ‘typical’ month (median) | 85.3 | 85.3 | 41.7 | 60.8 |
| Total number of patients ‘busy’ month (median) | 140.9 | 140.9 | 68.9 | 126.1 |
|  |  |  |  |  |
| Cost per patient |  |  |  |  |
| SoC Immunocompetent patients | € 85.99 | £ 72.03 | € 277.58 | € 127.85 |
| SoC Immunocompromised patients | € 102.34 | £ 85.73 | € 379.96 | € 211.11 |
| SoC All CAP patients^1^ | € 89.87 | £ 75.28 | € 323.65 | € 155.32 |
|  |  |  |  |  |
| Panel 1+3 strategy Immunocompetent patients | € 63.90 | £ 53.53 | € 177.98 | € 100.78 |
| Panel 1+3 strategy Immunocompromised patients | € 76.23 | £ 63.85 | € 271.18 | € 159.24 |
| Panel 1+3 strategy All CAP patients^1^ | € 66.83 | £ 55.98 | € 219.92 | € 120.07 |
|  |  |  |  |  |
| Average total cost per hospital per ‘typical’ month |  |  |  |  |
| SoC | € 7,666.04 | £ 6,421.28 | € 13,496.04 | € 9,443.71 |
| Panel 1+3 strategy | € 5,700.46 | £ 4,774.85 | € 9,170.71 | € 7,300.21 |
| Difference between SoC and Panel 1+3^2^ | -€ 1,965.58 | -£ 1,646.42 | -€ 4,325.33 | -€ 2,143.50 |
|  |  |  |  |  |
| Average total cost per hospital per ‘busy’ month |  |  |  |  |
| SoC | € 12,662.90 | £ 10,606.78 | € 22,299.21 | € 19,586.38 |
| Panel 1+3 strategy | € 9,416.12 | £ 7,887.19 | € 15,152.56 | € 15,140.73 |
| Difference between SoC and Panel 1+3^2^ | -€ 3,246.78 | -£ 2,719.59 | -€ 7,146.65 | -€ 4,445.64 |

CAP, community acquired pneumonia; SoC, standard of care. Baseline values from the model were used including the baseline cost for Panel 1 (€23.88 equivalent to £20.00) and for Panel 3 (€23.88 equivalent to £20.00).

^1^Weighted by percentage of immunocompetent and immunocompromised patients. ^2^A negative number here indicates that the Panel 1+3 strategy results in cost savings compared to SoC.

**Supplementary Table 16. Average cost per patient admitted with CAP if 100% were tested with both panel tests compared to SoC**

|  | **UK €** | **UK £** | **France** | **Spain** |
| --- | --- | --- | --- | --- |
| Immunocompetent patients |  |  |  |  |
| SoC | € 85.99 | £ 72.03 | € 277.58 | € 127.85 |
| Panel 1 | € 66.89 | £ 56.03 | € 245.75 | € 102.98 |
| Panel 3 | € 100.91 | £ 84.53 | € 197.10 | € 125.18 |
| Panel 1+3 | € 83.00 | £ 69.53 | € 177.98 | € 105.55 |
| Difference between SoC and Panel 1+3^1^ | -€ 2.98 | -£ 2.50 | -€ 99.60 | -€ 22.30 |
| Immunocompromised patients |  |  |  |  |
| SoC | € 102.34 | £ 85.73 | € 379.96 | € 211.11 |
| Panel 1 | € 87.42 | £ 73.23 | € 349.73 | € 177.55 |
| Panel 3 | € 104.88 | £ 87.85 | € 297.58 | € 189.55 |
| Panel 1+3 | € 92.94 | £ 77.85 | € 275.96 | € 160.43 |
| Difference between SoC and Panel 1+3^1^ | -€ 9.40 | -£ 7.88 | -€ 104.00 | -€ 50.68 |

SoC, standard of care; UK, United Kingdom.

The baseline cost of €23.88 (£20.00) per panel was used for Panel 1 and for Panel 3.

^1^A negative number here indicates that the Panel 1+3 strategy results in cost savings compared to SoC.

**Supplementary Figure 1. UK: Tornado plots showing impact of key parameters on average cost per patient for SoC and Panel 1+3 strategy**

Abbreviations: £, cost; % percentage; BAL, bronchoalveolar lavage, COVID-19, SARS-CoV-2; IF, immunofluorescence; PCR, polymerase chain reaction; SoC, standard of care; sp, species; UAT, urinary antigen test; UK, United Kingdom.

The shaded bars represent low values, and the partially shaded bars represent high values.

**Supplementary Figure 2. France: Tornado plots showing impact of key parameters on average cost per patient for SoC and Panel 1+3 strategy**

Abbreviations: €, cost; % percentage; BAL, bronchoalveolar lavage, COVID-19, SARS-CoV-2; IF, immunofluorescence; PCR, polymerase chain reaction; SoC, standard of care; sp, species; UAT, urinary antigen test.

The shaded bars represent low values, and the partially shaded bars represent high values.

**Supplementary Figure 3. Spain: Tornado plots showing impact of key parameters on average cost per patient for SoC and Panel 1+3 strategy**

Abbreviations: €, cost; % percentage; BAL, bronchoalveolar lavage, COVID-19, SARS-CoV-2; IF, immunofluorescence; PCR, polymerase chain reaction; SoC, standard of care; sp, species; UAT, urinary antigen test.

The shaded bars represent low values, and the partially shaded bars represent high values.
